# Supplementary material for: Do multidisciplinary cancer care teams suffer decision-making fatigue: an observational, longitudinal team improvement study
Source: BMJ Open. 2019 May 27;9(5):e027303. doi: 10.1136/bmjopen-2018-027303 (PMC6549703; doi:10.1136/bmjopen-2018-027303)
Supplement: Author's manuscript [file bmjopen-2018-027303.draft_revisions.pdf]

BMJ Open is committed to open peer review. As part of this commitment we make the peer review history of every article we publish publicly available.

When an article is published we post the peer reviewers' comments and the authors' responses online. We also post the versions of the paper that were used during peer review. These are the versions that the peer review comments apply to.

The versions of the paper that follow are the versions that were submitted during the peer review process. They are not the versions of record or the final published versions. They should not be cited or distributed as the published version of this manuscript.

BMJ Open is an open access journal and the full, final, typeset and author-corrected version of record of the manuscript is available on our site with no access controls, subscription charges or pay-per-view fees (http://bmjopen.bmj.com).

If you have any questions on BMJ Open's open peer review process please email info.bmjopen@bmj.com

Do multidisciplinary cancer care teams suffer decision-making fatigue? An observational, longitudinal team improvement study

Journal:	*BMJ Open*
Manuscript ID	bmjopen-2018-027303
Article Type:	Research
Date Submitted by the Author:	17-Oct-2018
Complete List of Authors:	Soukup, Tayana ; King's College London, Centre Implementation Science; King's College London<br>Gandamihardja, Tasha; Chelmsford Breast Unit<br>McInerney, Sue; Imperial College Healthcare NHS Trust<br>Green, James; Barts Health NHS Trust, Department of Urology, Whipps Cross University Hospital; London South Bank University<br>Sevdalis, Nick; King's College London,
Keywords:	Cancer multidisciplinary team meetings, decision-making, decision-making fatigue

SCHOLARONE™
Manuscripts

Do multidisciplinary cancer care teams suffer decision-making fatigue? An observational, longitudinal team improvement study

Tayana Soukup PhD[a✉], Tasha Gandamihardja MEd PhD FRCS[b], Sue McInerney RGN[c], James S. A. Green LLM FRCS (Urol)[ad], Nick Sevdalis PhD[a]

Affiliations List

[a] Centre for Implementation Science, King's College London, UK

[b] Chelmsford Breast Unit, Broomfield Hospital, Chelmsford, UK

[c] Imperial College Healthcare NHS Trust, Charring Cross Hospital London, UK

[d] Whipps Cross University Hospital, London, UK

Corresponding author

✉ Dr Tayana Soukup, King's College London, Health Services and Population Department,

Demark Hill, London SE5 8AF, E-mail: tayana.soukup@kcl.ac.uk Tel.: +44 20 7848 0683.

Short Title

Decision-making fatigue in oncology meetings

Keywords

Cancer multidisciplinary team meetings; decision-making; decision-making fatigue;

Word count

4424 words

For peer review only

ABSTRACT

Objective

The objective of this study was to examine effectiveness of co-designed quality-improving interventions with a multidisciplinary team (MDT) with high workload and prolonged meetings to ascertain: (1) presence and impact of decision-making (DM) fatigue on team performance in the weekly MDT meeting, and (2) impact of a short meeting break as a countermeasure of DM fatigue.

Design and interventions

This is a longitudinal multiphase study with a co-designed intervention bundle assessed within team audit and feedback cycles. The interventions comprised short meeting breaks, as well as change of room layout and appointing a meeting chair.

Setting and participants

A breast cancer MDT with 15 members was recruited between 2013 and 2015 from a teaching hospital of the London (UK) metropolitan area.

Measures

A validated observational tool (Metric for the Observation of Decision-making, MDT-MODe) was used by trained raters to assess quality of DM during 1,335 patient-reviews. The tool scores quality of information and team contributions to reviews by individual disciplines (Likert-based scores), which represent our two primary outcome measures.

Results

Data were analysed using multivariate analysis of variance. DM fatigue was present in the MDT meetings: quality of information (M=16.36 to M=15.10) and contribution scores (M=27.67 to M=21.52) declined from 1st to 2nd half of meetings at baseline. Of the improvement bundle, we found breaks reduced the effect of fatigue: following introduction of breaks (but not other interventions) information quality remained stable between 1st and 2nd half of meetings (M=16.00 to M=15.94), and contributions to team DM improved overall (M=17.66 to M=19.85).

Conclusion

Quality of cancer team DM is affected by fatigue due to sequential case-review over often prolonged periods of time. This detrimental effect can be reversed by introducing a break in the middle of the meeting. The study offers a methodology based on 'team audit and feedback' principle for co-designing interventions to improve teamwork in cancer care.

For peer review only

STRENGTHS

1. A validated tool was used
2. Subset of cases was scored by trained evaluators in pairs blind to one another's scores
3. Main assessor was a clinician whose presence in MDT meetings is natural

LIMITATIONS

1. Observer bias and Hawthorne effect
2. Pre-post study design with no control over extraneous elements that are changing at the same time as the intervention is implemented (this is because MDT meetings are mandatory in the UK, and randomised controlled trials are not possible)

For peer review only

INTRODUCTION

In the UK, care planning for patients with cancer is routinely (and mandatorily) carried out by a multidisciplinary team (MDT), generally consists of histopathologists, radiologists, surgeons, specialist cancer nurses and oncologists, in typically weekly meetings (MDMs, or tumour boards). Here, patients are reviewed and treatment recommendations are agreed upon by the team in a sequential manner for up to a few hours at a time.[1-5,7-8] While the MDT approach to cancer care is endorsed widely,[7] evidence of its effectiveness is unclear and variable.[8-17] A pattern generally observed in MDMs is unequal participation to discussion and suboptimal sharing of information.[1,8-16] Evidence from studies on small groups suggests that variability in performance is attributable to human factors, such as those that are internal to teams incl. leadership, group composition and personality traits, as well as the external circumstances, such as increasing workload, time pressures, and shifting economic landscape.[18]

Hence one aspect of MDMs warrants further focus, and that is the type of fatigue that arises as a result of increasing workload. To-date, evidence has documented high workloads on cancer MDTs with meetings up to 5h reported in the recent Cancer Research UK report.[5] For example, in the UK, studies have reported that a breast cancer MDT reviewed between 29 and 51 patients with the meeting often running for up to 3.5h;[1] lung MDT between 22 and 30 patients with meetings up to 3h;[2] urology MDT between 19 and 51 patients with meetings up to 2h;[3] and a colorectal MDT between 9 and 55 patients with meetings up to 1h and 40min.[4] High workloads and prolonged periods of consecutive DM in the meetings have become a norm for many teams,[6,8] something that is likely to continue as teams are trying to maximise productivity in the face of increasing numbers of new cancer cases worldwide,[19-20] rising financial pressures,[20-21] and growing staff shortages.[22]

Little is known however about the impact of such intense periods of cognitive activity on clinical performance, in particular in cancer MDMs, with one study showing that the quality of endoscopy performance declines with successive procedures.[36] Evidence from cognitive science shows that such consecutive efforts can lead to cognitive depletion, negatively affecting subsequent decisions, leading to performance decrements over time – also known as *decision-making fatigue (DM fatigue)*.[23-24] Consequences are many, including: rushed decisions, lack of attention to all available information and potential implications, status quo,[25-26] reduced ability to effectively evaluate choices and sustain attention, as well as easy distractibility and absentmindedness.[27-29] Strategies,

For peer review only - http://bmjopen.bmj.com/site/about/guidelines.xhtml

such as short breaks, consuming food, glucose and water can help safeguard against decision fatige,[24,30-35] something that in other industries, such as aviation, has been recognisied.[34-35]

This is not the case for healthcare, however. On the one hand, the World Health Organisation[37] recognises general fatigue as a leading contributor to medical error, and European Working Time Directive[45] restricts excessive night work and working hours. On the other hand, the type of fatigue that arises because of intensity and complexity of workload during working hours has not received the same level of recognition; despite healthcare being fraught with examples of intense cognitive work.[38-40] To-date, the impact of DM fatigue has not been explored in healthcare settings; our objective was to examine this concept for the first time within the current study design.

One way of testing and evaluating the concept of DM fatigue with an MDT is to apply the principles of 'team audit and feedback' - a process of providing non-punitive and actionable feedback to professionals to allow them to self-assess and adjust their performance, thus stimulating desired behaviour change.[41-42] Such approach was found effective in improving practice and supporting quality improvements, and can be used to aid implementation of evidence-based interventions.[41] Within our study, this approach allowed us to elicit inputs from all team members, which we then used to co-design interventions to best meet the needs of the team in addressing DM fatigue. As a team-centred approach to intervention development, implementation and evaluation, this is, to the best of our knowledge, yet to be applied to cancer MDTs.

Aim and objectives

Following on from our feasibility study,[1] we examined effectiveness of co-designed quality-improving interventions with an MDT with high workload and prolonged meetings as a concrete setting to ascertain: (1) presence and impact of DM fatigue on team performance in MDMs, and (2) impact of a short break in MDMs as a countermeasure of DM fatigue.

METHODS

Study design

This was a longitudinal prospective observational study carried out over a 2-year period with a breast cancer MDT. Interventions were introduced within a single arm pre-post study design.

Patient and public involvement

Patients and public were not involved in the development and design of this study.

For peer review only

Setting

A breast cancer MDT was recruited between 2013 and 2015 from a teaching hospital of the London (UK) metropolitan area.

Participants

Participants were 15 members of a breast cancer team, and a total of 1,335 breast cancer patients reviewed at 30 MDMs. Availability sampling was used to identify the team with a criterion for the study being a cancer MDT from the UK National Health Service (NHS) that represents one of the most common types of cancer, and experiences high workload with prolonged meeting duration (>1h). Sample size in terms of number of MDMs per study phase (*n*=10) was determined based on our feasibility study,[1] and a prior study of our group in urology with similar workload.[12] The study was granted Ethical Approval by the local ethics committee (JRCO REF. 157441).

Intervention Design: Audit and Feedback Cycles

Interventions were co-designed and evaluated based on the principles of team audit and feedback.[41-42] In what follows, we outline what this process entailed.

Audit cycles focused on collecting observational data of team DM processes across 3 phases. In **phase 1** (baseline; MDMs 1 to 10; July to Nov 2013), we did not introduce any interventions, but observations of care as usual. The descriptive data from this phase have been reported as a pilot study to establish feasibility of the measurement.[1] In **phase 2** (MDMs 11 to 20; Feb to April 2014), we introduced two interventions including (1) *change of the room layout from lecture theatre style to a U-shape* where team-members were able to face each other, and (2) formal appointment of an *MDM chair-person*. The rationale for these interventions was that the change of the room layout will be more conducive to team interactions, while appointment of the formal chair will help facilitate the overall flow of the meeting and individual patient discussions. In the final **phase 3** (MDMs 21 to 30; Sept 2014 to March 2015), we introduced a 10-minute long break for tea, coffee and snacks halfway through the MDM, i.e., typically at the 90-minute mark, which was hypothesised to help counteract negative effects of DM fatigue.

Feedback sessions focused on co-designing interventions. They occurred at 3 time points at the end of each audit phase – in June 2014, May 20014 and June 2015. Each session was allocated a 1h slot as part of the MDM where we **(a)** fed back the summary of the analysis (20-minutes), **(b)**

For peer review only - http://bmjopen.bmj.com/site/about/guidelines.xhtml

facilitated team-based review of the findings and what they meant for the team (20-minutes); and **(c)** shortlisted evidence-based interventions the team were willing to introduce into their work in the coming study period (20-minutes).

Following the feedback sessions, the research team produced minutes and actions, which were approved and emailed to the MDT by their lead, a Consultant Breast Surgeon (TG). The MDT was invited to comment and identify date for intervention implementation. The task of leading the introduction of the interventions was assigned to the MDT lead. Interventions were introduced and allowed a 'bed-in' period of approximately 3 months, during which no assessments were carried out to allow the team to familiarise themselves with the novel way of working. This approach was designed at the request of the MDT who needed the 'bed-in' time to ensure they did not feel they were being 'examined' by the research team at a time when they were in a state of change.

Materials

We used a validated quantitative observational assessment tool, namely the Metric for Observation of Decision-making, (MDT-MODe; Figure 1),[10] which was tested for feasibility in our pilot study.[1] The tool has been used previously to assess various cancer MDMs and has shown good validity and reliability.[1-4,10-14] It consists of two domains – (1) quality of presented patient information and (2) quality contribution to case-discussion. The tool is described in detail in our feasibility study.[1]

Figure 1

Assessor training

Prior to the formal scoring during the study, the evaluator (Cancer Nurse Specialist, SM) was trained in the use of the MDT-MODe.[10] Training is essential to be able to use it, which is a general principle for instruments assessing human factors in clinical environments.[44] Training was delivered by our team and it involved: (1) explanation of the domains, scales and their anchors, (2) background reading of peer-reviewed literature on the tool, and (3) calibration of scoring against an expert evaluator (TS) via scoring a set of pre-recorded MDT videos.

To ensure reliability in the use of the tool, a cross-section of the data was double-rated blindly by trained clinical (SM) and psychologist (TS) observers. To minimise H*awthorne effect*, i.e., teams changing their usual behavior due to being observed, the main study evaluator was the Cancer

For peer review only - http://bmjopen.bmj.com/site/about/guidelines.xhtml

Nurse Specialist, the presence of whom within an MDM is natural. During data collection, each evaluator was blind to the other evaluators' observations, and the observer (SM) did not participate in the MDMs clinically. Proficiency in scoring was set as an achievement of inter-assessor reliability of 0.70 or higher between the trainee and expert assessor;[44] this was met.

Statistical methods and variables

There were two independent variables (IVs) in the study:

- ❖ IV1 was defined as the '*study phase*' with 3 levels (phases 1, 2 and 3) in the one-way multivariate analysis, and 2 levels (phases 2 and 3) in the two-way multivariate analysis;
- ❖ IV2 was defined as the '*time lapse*' with 2 levels, namely, 1st and 2nd half of the meeting.

There were two dependent variables (DVs):

- ❖ DV1 is *quality of presented patient information* to the team as measured by MDT-MODe,[10,1]
- ❖ DV2 is *quality of disciplinary contributions to patient-review* as measured by MDT-MODe[10,1]

Three sets of analyses were conducted:

1. *Intra-class correlation coefficient (ICC)* analysis was used to assess reliability of evaluations in each phase. ICCs can range between 0 and 1, with higher values indicating better agreement.
2. *Multivariate analysis of variance (MANOVA*) was used to assess:
   a. *between-intervention differences* in DM where the effect of co-designed interventions across all 3 phases is explored using a *one-way MANOVA* with *post-hoc tests*;
   b. *within-meeting differences* in DM where presence of DM fatigue and effect of a 10-min break in phases 2 and 3 is explored using *two-way MANOVA* with *simple main effects*.
3. *Correlation analysis* was used to ascertain presence of DM fatigue across all 3 phases.

All analyses were carried out using SPSS® version 20.0. All pairwise comparisons are reported with Bonferroni-adjusted *p*-values.

Data sharing statement

The anonymised data set[45] supporting this study is available on Zenodo, a research data repository, under the Creative Commons Attribution Non-Commercial Non-Derivative 4.0 license. The researchers are free to reuse and redistribute the data set[45] on the condition that they *attribute it*, that they *do not use it for commercial purposes*, and that they *do not alter it*. For any reuse or redistribution, researchers must make clear to others *the license terms* of this work.

For peer review only - http://bmjopen.bmj.com/site/about/guidelines.xhtml

RESULTS

Meeting characteristics

The sample consisted of overall 1,335 patients managed across the 3 study phases (see Table 1). It is evident that the total number of patients discussed per phase steadily increased as the study progressed, which suggests increasing workload for the team over time.

Table 1

Reliability of evaluations

Agreement between evaluators was assessed on a randomly selected subset of patient-reviews within each phase using single measures interclass correlation with the two-way mixed effects model and an absolute agreement definition. High reliability was obtained across all stages:

- *Baseline/Phase 1*: information $r = 0.89$, contribution $r = 0.82$, $n = 116$, 34% of the cohort;
- *Phase 2*: information $r = 0.92$, contribution $r = 0.95$, $n = 116$, 25% of the cohort;
- *Phase 3*: information $r = 0.88$, contribution $r = 0.79$, $n = 131$, 25% of the cohort.

Between-intervention differences in decision-making across all 3 phases

A one-way ANOVA was run to determine effect of co-designed interventions (IV1 with 3 levels: phases 1, 2 and 3) on the information (DV1) and contribution (DV2) scores of the MDT-MODe.[10] Data are expressed as mean ± standard deviation. To preserve statistical power, Bonferroni adjusted p-level of 0.025 was used.

Information scores were similar between phase 1, 2 and 3 (16.31±3.71; 15.76±2.98 and 15.97±3.77, respectively), while the *contribution scores* were lower in phase 1 than 2 and 3 (17.16±3.23; 22.13±3.40; 18.81±5.50, respectively). There is statistically significant difference between the intervention phases on the *combined DVs*, $F(4, 2664)= 76.49$, $p<0.001$; Pillai's Trace=0.10; partial $\eta^2=0.10$.

Follow-up univariate ANOVAs showed that the *information scores* alone did **not** reveal significant differences between phases ($F(2, 1332)=2.44$, $p=0.09$; partial $\eta^2 =0.004$), while the *contribution scores* **did** ($F(2, 1332)=144.69$, $p<0.025$; partial $\eta^2 =0.18$). Bonferroni post-hoc tests revealed that for *contribution scores*, phase 2 had significantly higher mean score than phases 1 ($p<0.02$) and 3 ($p>0.02$); and that phase 3 had significantly higher mean score than phase 1 ($p<0.02$).

For peer review only - http://bmjopen.bmj.com/site/about/guidelines.xhtml

In sum, the findings show that the *quality of information* remained largely similar across phases, while the *quality of contribution* improved in phases 2 and 3 relative to phase 1 but with no linear improvement across phases. See figure 2 (a and b) for a graphical representation of the results.

Figure 2

Within-meeting differences in decision-making in phases 2 and 3

A two-way MANOVA was conducted to examine interaction effects between *IV1 or a 10-minute break* (two levels: phase 2 meetings with no break, and phase 3 meetings with a break), and IV2 or '*time lapse'* (two levels: 1st and 2nd half of meetings) on the information (DV1) and contribution (DV2) scores of the MDT-MODe.[10] Data are expressed as mean ± standard deviation.

There was a significant interaction effect between *10-minute break* and *time lapse* on the *information*, $F(1, 984)=5.21$, $p<0.01$, partial $\eta^2 =0.01$, and *contribution scores,* $F(1, 984)=45.55$, $p<0.001$, partial $\eta^2 =0.03$. An *analysis of simple main effects* for a *10-minute break* and *time lapse* was performed with significance Bonferroni-adjusted for $p<0.0125$. There was a significant difference in mean *information scores* for 1st v. 2nd half of the meeting in phase 2, $F(1, 985) =16.00$, $p<0.001$, partial $\eta^2 =0.02$, and a non-significant difference in phase 3 when the meeting break was introduced, $F(1,985) =0.04$, $p=0.845$, partial $\eta^2 =0.00$. There was also a significant difference in mean *contribution scores* for 1st v. 2nd half of the meeting in phase 2, $F(1, 985)=7.44$, $p<0.01$, partial $\eta^2 =0.01$, and also in phase 3, $F(1, 985)=30.23$, $p<0.001$, partial $\eta^2 =0.03$.

Follow-up pairwise comparisons were run for each simple main effect with reported 95% confidence intervals and *p*-values Bonferroni-adjusted to 0.0125. See Table 2 below for the results, and Figure 2 above for a graphical representation of the comparisons reported here.

Table 2

In sum, *quality of information and contribution* was reduced in the 2nd half of the meeting when the MDT did not have a 10min break (phase 2). In contrast, when the MDT had a break (phase 3), the quality of information remained unchanged, while the quality of contribution improved.

For peer review only - http://bmjopen.bmj.com/site/about/guidelines.xhtml

Correlation analysis: ordinal position of cases and quality of dm across study phases

A follow-up analysis was conducted on the ordinal position of cases within meetings, and information and contribution scores *to ascertain* performance decrements across all 3 phases, and improvements obtained in phase 3 because of a 10-minute break. Ordinal position of a case within an MDM is taken as an indicator of potential effects of DM fatigue: the later a case is reviewed during the MDM, the more cases the team would have reviewed in a sequential manner prior to it.

Table 3 shows significant negative correlations between ordinal position of cases, and contribution and information scores in phases 1 and 2 - i.e., as the ordinal position of cases increases (i.e., the patient is reviewed later in the meeting), the information and contribution scores decrease (i.e., team interaction and clinical input measures worsen). In phase 3, however, when the short break was introduced, both coefficients are non-significant, indicating overall improvement – i.e., a lack of impact of the repetitive DM process on the team interaction and clinical input indicators.

Table 3

Table 3 also shows that the intervention package introduced in phase 2 (change of room layout and appointing a meeting-chair) did not influence the quality of DM when assessed within meetings; these effects are only detectable in the between-intervention analysis (Figure 2a and 2b).

Team's feedback on the conduct of the meetings

In the final feedback session (June 2015), the team recognised that the meeting break and seating rearrangement were useful and had positive impact on their working, while appointing a rotating chairperson presented with challenges and is something that would need more focus in order to ensure consistency across weekly meetings. The team reported two reasons for this, one, team friction and lack of clarity around who is chairing, and second, fatigue that the chairperson experiences by having to chair the meeting and contribute clinically to discussion ('chairing fatigue'). The team proposed that, going forward, this could be addressed by assigning the chairing role to another member of the team in the 2nd half of the meeting.

For peer review only - http://bmjopen.bmj.com/site/about/guidelines.xhtml

DISCUSSION

The overall aim of this study was to examine effectiveness of co-designed interventions with a breast cancer team with high workload and prolonged meeting duration, and within this, explore presence and impact of DM fatigue, and a short break as a countermeasure. Our findings were threefold. Firstly, our study lends support for the concept of DM fatigue in MDMs.[23-24] In phase 2, the information and contribution quality were significantly lower in the 2nd v. 1st half of the meeting. The serial position of cases in the meetings in phases 1 and 2 were also negatively correlated with information and contribution quality, indicating performance decrements as meetings progressed. However, after the break was introduced in phase 3, serial position of cases no longer showed significantly negative correlation with information and contribution quality, lending support to a premise that short break in the middle of a meeting can counterbalance the effect of DM fatigue (our second finding).[24,30-35]

Thirdly, we found a significant increase in information and contribution quality after the introduction of interventions in phases 2 and 3 in comparison to baseline (or, phase 1). This somewhat lends support to co-designed interventions via audit and feedback.[41-43] However, a significant decrease was evident in phase 3 in comparison to phase 2, pointing to challenges at sustaining initially implemented interventions over time. In line with the final team's feedback, one explanation may be chairing fatigue and team friction, which highlights the need for continuous quality improvements and implementation science approaches to help improve our understanding of barriers and facilitators to the uptake of evidence-based interventions for cancer MDTs. It is possible that the feedback should be provided to the team at shorter intervals (after every 5th as opposed to every 10th meeting) to help reinforce the agreed change and goals. Another element that could have (also) indirectly contributed to these findings is the steady increase in workload across phases (Table 2), which is known to negatively impact MDT-working.[16-17]

Nonetheless, despite the nonlinear trajectory between phases 2 and 3, the improvements were made in the within-meeting performance i.e. between 1st and 2nd half of the meeting in phase 3 after the 10-minute break was introduced. This lends support to the concept of DM fatigue - i.e., fatigue that arises because of consecutive cognitive efforts in formulating treatment recommendations, previously explored in other fields (e.g. judicial DM).[24-25] Improved quality of discussion between different disciplines is observed when break is introduced with the quality of presented patient

For peer review only - http://bmjopen.bmj.com/site/about/guidelines.xhtml

information becoming more stable throughout the meeting. What is more, the 10-minute break did not add additional time to the meeting duration (Table 1), indicating that taking a break made the team more time efficient. The concept of DM fatigue has not yet been explored within cancer MDMs, and to our knowledge this is the first study of its kind, with implications for the way meetings are currently structured.

Implications

The implications for meeting structure are far-reaching. It is not only the number of hours worked in a 24-hour period, but also the number of consecutive hours, including the type, intensity and complexity of a task, a clinician engages in without adequate break that requires more focus and recognition. Healthcare is a highly demanding work setting, and apart from MDMs, there are many examples of cognitively intense settings, including for e.g., ward rounds and intensive care units.[38-39] While the general health worker fatigue is addressed by the European Working Time Directive[46] which restricts excessive night work and working hours, the type of fatigue that arises as a result of intensity and complexity of the workload during the working hours is not adequately acknowledged or safeguarded with recommendations, such as a short break, for instance. It is understood however that the fatigue is a leading contributor to medical error and injury,[37] and that intense episodes of workload in healthcare are on the increase,[19-21,5] as clinical teams are trying to maximise productivity in the face of severe staff shortages[22] and financial pressures.[20-21]

Limitations

Our findings need to be interpreted within certain limitations (some of which have been previously reported).[1] First, participants in our study were aware that they were being observed, hence we cannot rule out observer bias and *Hawthorne effect.* We addressed the former by using a validated tool with a subset of cases scored by *trained* evaluators in pairs who were blind to one another's observations during the study. In terms of the latter, which is a natural limitation to observational studies, we ensured that the main study evaluator was a clinician, in our case, Cancer Nurse Specialist, the presence of whom within an MDM is natural.

Second, while this is a large-scale study for its nature (observations in real-time), we acknowledge that there are cancer MDMs that are not as long as the ones reported here, hence the *generalizability of our findings may be limited* to MDTs with high workloads and prolonged meeting duration within the NHS setting. However, the global economic and healthcare landscape

For peer review only

is rapidly changing – i.e. cancer incidence[19-20] is on the increase, as well as MDT workload,[5,19] financial pressures,[20-21] and staff shortages.[22] The findings that we report may therefore become increasingly relevant to MDTs across different tumor types (and other healthcare settings) globally and could be profitably explored to determine the extent to which they apply to them.

Third, our study is of *pre-post design*, which can limit generalizability of our findings. This is because there is no control over other (extraneous) elements that are also changing at the same time as the intervention is implemented. Since MDMs are mandatory in the UK, a randomised trial design and an arm with no intervention (i.e., no MDMs) are not possible. This makes the pre-post design most appropriate and feasible, in particular the observational approach, which is non-intrusive and does not add to team's workload. Such approach has allowed us to capture complex organisational behaviour of cancer MDMs in real time, providing good external validity and identifying new avenues of research.

Lastly, the validated tool used in the current study (MDT-MODe) *does not allow for individual person-level assessment*; only disciplinary group-level with the unit of analysis being a case-discussion (and not an individual team member; Figure 1). Such approach has advantages when evaluating a relatively small (single) team because it ensures team safety by minimizing the risk of defensive routine and blaming a particular team member for performance difficulties which could in turn distract the team from addressing their performance problems constructively.[47] We acknowledge however that such approach also has limitations because it does not capture (the effect of) team interaction, as well as (the effect of) individual team member's level of seniority, experience, and personality, and so the effect of the physician versus the team, or style of presentation of different radiologists/histopathologists cannot be accounted for. To address these questions, a different methodological approach may be better suited, such as Conversation Analysis for instance, which allows for an in-depth analyses of team interaction on an individual person-level. Also, development of tools for MDTs should take this limitation into account.

Further research

The objective of our study was to investigates presence and impact of fatigue on *DM processes* in a team with high workload; as such, we did not address how it impacts the *quality of decisions reached* (e.g., their clinical suitability for the patient) or *patient outcomes*. This is however an important next step that should be further explored in the light of our findings and previous

For peer review only - http://bmjopen.bmj.com/site/about/guidelines.xhtml

research showing that DM fatigue leads to impulsive decisions, status quo, and reduced ability to effectively evaluate information – these could potentially have a knock-on effect on patient outcomes.[17-22] Further research is also needed to assess presence of DM fatigue across different cancer MDTs, particularly those with high workloads, and explore effectiveness of various evidence-based cognitive strategies.[24,30-33] Efforts should be channelled toward safeguarding optimal DM in MDMs, taking into account the intensity and complexity of the workload, with strategies in place as standard practice - such as, for instance, a maximum limit of cases allowed for a single meeting, mandatory short break (as practiced in the aviation industry), and trained team lead/chairperson to help the team effectively navigate through workload.[6] Team-centred, co-designed approaches may prove useful in helping identify appropriate (tailored) strategies for a team, however, challenges exist at sustaining change over time; hence, a need for continuous quality improvement and implementation science approaches in the field of cancer MDTs.

CONCLUSIONS

Previous research has shown variability in the quality of DM across cancer MDMs, with internal factors, such as group composition and leadership, and external circumstances, such as increased workload, time pressures and changing economic landscape held accountable. Our study demonstrates for the first time that quality of DM in cancer MDMs grows worse during consecutive cognitive efforts and is positively influenced with a break. Using principles of team audit and feedback to co-design team-centred interventions is a useful approach in helping initiate improvements, however, challenges exist at sustaining interventions over time. Building on our findings, further research in MDTs is needed to investigate effects of DM fatigue on the quality of decisions reached and patient outcomes, ascertain its presence across different cancer teams, and encourage implementation of quality-improving strategies to protect optimal DM. The work could be extrapolated to other areas of clinical (and non-clinical) practice and may have implications for other areas that have equally intense periods of cognitively demanding work.

ACKNOWLEDGEMENTS

The authors would like to thank the breast cancer MDT and their members for their time and commitment to this project.

For peer review only

DISCLOSURE

Nick Sevdalis is the Director of London Safety & Training Solutions Ltd, which provides team working, patient safety and improvement skills training and advice on a consultancy basis to hospitals and training programs in the UK and internationally. James Green is a Director of Green Cross Medical Ltd that developed MDT FIT for use by National Health Service Cancer Teams in the UK. The other authors have no conflicts of interest to report.

FUNDING

Financial support for this study was provided entirely by the UK's National Institute for Health Research (NIHR) via the Imperial Patient Safety Translational Research Centre. Sevdalis and Soukup's research is funded by the NIHR via the 'Collaboration for Leadership in Applied Health Research and Care South London' at King's College Hospital NHS Foundation Trust, London, UK. Sevdalis is also a member of King's Improvement Science, which is part of the NIHR CLAHRC South London and comprises a specialist team of improvement scientists and senior researchers based at King's College London. Its work is funded by King's Health Partners (Guy's and St Thomas' NHS Foundation Trust, King's College Hospital NHS Foundation Trust, King's College London and South London and Maudsley NHS Foundation Trust), Guy's and St Thomas' Charity, the Maudsley Charity and the Health Foundation. The funding agreement ensured the authors' independence in designing the study, interpreting the data, writing, and publishing the report. The views expressed are those of the authors and not necessarily those of the NHS, the NIHR or the Department of Health and Social Care.

For peer review only

AUTHORS' CONTRIBUTIONS

In line with the guidelines by the International Committee of Medical Journal Editors, all authors for this study (i.e., TS, TG, SM, SM, JG, and NS) have made substantial contributions to conception and design, or acquisition of data, or analysis and interpretation of data; have been involved in drafting the manuscript or revising it critically for important intellectual content; have given final approval of the version to be published; and have agreed to be accountable for all aspects of the work in ensuring that questions related to the accuracy or integrity of any part of the work are appropriately investigated and resolved.

For peer review only - http://bmjopen.bmj.com/site/about/guidelines.xhtml

REFERENCES

1 Gandamihardja TAK, Soukup T, McInerney S, Green JSA, Sevdalis N. (in press) Analyzing breast cancer multidisciplinary patient management: a prospective observational evaluation of team clinical decision making. *World J Surg* 2018;in press.

2 Sarkar S, Arora S, Soukup T, Lamb BW, Shah S, Green JSA, Sevdalis N, Darzi, A. A multi-centre study evaluating performance of multidisciplinary teams: Urology vs the top cancer killers. *Eur Urol Suppl* 2014;13: e878-e878a.

3 Lamb BW, Sevdalis N, Benn J, Vincent C, Green JS. Multidisciplinary cancer team meeting structure and treatment decisions: A prospective correlational study. *Ann Surg Onc* 2013;20: 715–722.

4 Jalil R, Akhter W, Lamb BW, Taylor C, Harris J, Green JSA. Validation of team performance assessment of multidisciplinary tumor boards. *J Urol* 2014;192(3): 891-898.

5 Cancer Research UK. Improving the effectiveness of multidisciplinary team meetings in cancer services. London, Cancer Research UK; 2017.

6 National Cancer Action Team. The characteristics of an effective multidisciplinary team (MDT). London, UK: National Cancer Action Team; 2010.

7 Department of Health. Manual for Cancer Services. London, UK: The Department of Health; 2004.

8 Raine R, Xanthopoulou P, Wallace I. Determinants of treatment plan implementation in multidisciplinary team meetings for patients with chronic diseases: a mixed-methods study. *BMJ Qual Saf* 2014;23: 867–876.

9 Hong NJ, Wright FC, Gagliardi AR, Paszat LF. Examining the potential relationship between multidisciplinary cancer care and patient survival: An international literature review. *J Surg Oncol* 2010;102: 125-134.

10 Lamb BW, Wong HWL, Vincent C, Green JSA, Sevdalis N. Teamwork and team performance in multidisciplinary cancer teams: Development of an observational assessment tool. *BMJ Qual Saf* 2011;20: 849-856.

11 Lamb BW, Brown K, Nagpal K, Vincent C, Green JS, Sevdalis N. Quality of care management decisions by multidisciplinary cancer teams: A systematic review. *Ann Surg Oncol* 2011;18: 2116-2125.

For peer review only

12 Lamb BW, Green JS, Benn J, Brown KF, Vincent C, Sevdalis N. Improving decision making in multidisciplinary tumor boards: Prospective longitudinal evaluation of a multicomponent intervention for 1,421 patients. *J Am Coll Surg* 2013;217(3): 412-420.

13 Soukup T, Lamb BW, Sarkar S, Arora S, Shah S, Darzi A, Green JSA, Sevdalis N. Predictors of treatment decision in multidisciplinary oncology meetings: A quantitative observational study. *Ann Surg Oncol* 2016;23(13): 4410-4417.

14 Soukup T, Petrides KV, Lamb BW, Sarkar S, Arora S, Shah S, Darzi A, Green JSA, Sevdalis N. The anatomy of clinical decision-making in multidisciplinary cancer meetings: A cross-sectional observational study of teams in a natural context. *Medicine* 2016;95;e3885.

15 Stairmands J., Signal L, Sarfati D, Jackson C, Batten L, Holdaway M, Cunningham C. Consideration of comorbidity in treatment decision-making in multidisciplinary team meetings: A systematic review. *Ann Oncol* 2015;26(7): 1325-1332.

16 Kidger J, Murdoch J, Donovan JL, Blazeby JM. Clinical decision-making in a multidisciplinary gynaecological cancer team: a qualitative study. *BJOG* 2009;116(4): 511-517.

17 Haward R, Amir Z, Borrill C, Dawson J, Scully J, West M, et al. Breast cancer teams: The impact of constitution, new cancer workload, and methods of operation on their effectiveness. *Br J Cancer* 2003;89(1): 15-22.

18 Poole MS, Hollingshead AB. Theories of small groups: Interdisciplinary perspectives. Thousand Oaks, CA: Sage; 2005.

19 Mistry M, Parkin DM, Ahmad AS, Sasieni P. Cancer Incidence in the UK: Projections to the Year 2030. *Br J Cancer* 2011;105: 1795-1803.

20 World Health Organization. World Cancer Report 2014. France: International Agency for Research on Cancer, World Health Organization; 2014.

21 NHS England. Everyone Counts: Planning for Patients 2014/2015 to 2018/2019. England, UK: NHS England; 2014.

22 NHS Improvement. Evidence from NHS Improvement on clinical staff shortages: A workforce analysis. London, NHS Improvement; 2016.

23 Baumeister RF. Ego depletion and self-control failure: An energy model of the self's executive function. *Self Identity* 2002;1: 129-136.

For peer review only

For peer review only - http://bmjopen.bmj.com/site/about/guidelines.xhtml

24 Danziger S, Levav J, Avnaim-Pesso L. (2012). Extraneous factors in judicial decisions. *Proc Natl Acad Sci* 2012;108(17): 6889-6892.

25 Kool W, McGuire JT, Rosen ZB, Botvinick MM. Decision making and the avoidance of cognitive demand. *J Exp Psychol* 2010;139(4): 665-682.

26 Eidelma S, Crandall CS. Bias in favour of the Status Quo. *Soc Personal Psychol Compass* 2012;6(3): 270-281.

27 Weissman DH, Roberts KC, Visscher KM, Woldorff MG. The neural bases of momentary lapses in attention. *Nat Rev Neurosci* 2006;9: 971-978.

28 Kaplan S, Berman MG. Directed attention as a common resource for executive functioning and self-regulation. *Perspect Psychol Sci* 2010;5: 43-57.

29 Sawin DA, Scerbo MW. Effects of instruction type and boredom proneness in vigilance: Implications for boredom and workload. *Hum Factors* 1995;37(4): 752-765.

30 Tyler JM, Burns KC. After depletion: The replenishment of the self's regulatory resources. *Self Identity* 2008;7: 305–321.

31 McMahon AJ, Scheel MH. Glucose promotes controlled processing: Matching, maximising and root beer. *Judgm Decis Mak* 2010;5(6): 450-457.

32 Kempton MJ, Ettinger U, Foster R, Williams SC, Calvert GA, Hampshire A, et al. Dehydration affects brain structure and function in healthy adolescents. *Hum Brain Mapp* 2011;32(1): 71-9.

33 Hagger MS, Chatzisarantis NL. The sweet taste of success: the presence of glucose in the oral cavity moderates the depletion of self-control resources. *Pers Soc Psych Bull* 2013;39: 28–42.

34 Neri DF, Shappell SA, DeJohn CA. Simulated sustained flight operations and performance, Part 1: Effects of fatigue. *Mil Psychol* 1992;4(3): 137-155.

35 Neri DF, Oyung RL, Colletti LM, Mallis MM, Tam PY, Dinges DF. Controlled breaks as a fatigue countermeasure on the flight deck. *Aviat Space Environ Med* 2002;73(7): 654-664.

36 Harewood GC, Chrysostomou K, Himy N, Leong WL. Impact of operator fatigue on endoscopy performance: Implications for procedure scheduling. *Dig Dis Sci* 2009;54(8): 1656–1661.

For peer review only

For peer review only - http://bmjopen.bmj.com/site/about/guidelines.xhtml

37 World Health Organisation. WHO Patient Safety Curriculum Guide for Medical Schools. France, A World Alliance for Safer Health Care; 2009.

38 Reader TW, Cuthberson BH, Decruyenaere J. Burnout in the ICU: Potential consequences for staff and patient well-being. *Intensive Care Med* 2008;34(1): 4-6.

39 Reader TW, Reddy G, Brett SJ. Impossible decision? An investigation of risk trade-offs in the intensive care unit. *Ergonomics* 2017;61: 122-133.

40 Reid PP, Compton D, Grossman JH, Fanjiang G. Building a better delivery system: A new engineering/health care partnership. Washington, D.C.: The National Academies Press; 2005.

41 Ivers N, Jamtvedt G, Flottorp S, Young JM, Odgaard-Jensen J, French SD. Audit and feedback: effects on professional practice and health care outcomes, Cochrane Database Sys Rev, no. 2, CD000259; 2006.

42 Flottorp SA, Jamtvedt G, Gibis B, McKee M. Using audit and feedback to health professionals to improve the quality and safety of health care. Denmark: World Health Organization on the behalf of the European Observatory on Health Systems and Policies; 2010.

43 Michie S, Johnston M, Abraham C, Lawton R, Parker D, Walker A. Making psychological theory useful for implementing evidence-based practice: a consensus approach. *Qual Saf Health Care* 2005;14: 26-33.

44 Hull L, Arora S, Symons NR, Jalil R, Darzi A, Vincent C. Delphi expert consensus panel. Training faculty in nontechnical skill assessment: national guidelines on program requirements. *Ann Surg* 2013;258(2): 370-375.

45 Soukup, T. Longitudinal intervention in a breast cancer team [Data set]. Zenodo 2018; http://doi.org/10.5281/zenodo.582198

46 The European Parliament and the Council of the European Union. Directive 2003/88/EC of the European Parliament and of the Council of 4 November 2003 concerning certain aspects of the organisation of working time. *Official Journal of the European Union*; 2013.

47 West MA. Effective teamwork: practical lessons from organisational research. John Wiley & Sons; 2012.

For peer review only

For peer review only - http://bmjopen.bmj.com/site/about/guidelines.xhtml

FIGURE CAPTIONS

Figure 1. Metric for the observation of decision-making in cancer multidisciplinary team meetings (MDT-MODe)

Figure 2. Mean scores for information and contribution quality across the observational phases 1, 2 and 3, as well as across the 1st and 2nd half of the meetings in phases 2 and 3 only

For peer review only

TABLES

For peer review only

For peer review only - http://bmjopen.bmj.com/site/about/guidelines.xhtml

Table 1. Meeting characteristics of the breast cancer team across the intervention phases

**Meeting characteristics**	**Phase 1**				**Phase 2**				**Phase 3**			
	Total	Mean	Min	Max	Total	Mean	Min	Max	Total	Mean	Min	Max
Number of meetings observed*	10	-	-	-	10	-	-	-	10	-	-	-
Number of patients per meeting**	346	42	29	51	467	55	44	73	522	62	52	70
Time per patient-review (MM:SS)	-	03:20	00:31	09:00	-	03:00	00:47	09:06	-	02:06	00:10	12:49
Meeting duration (HH:MM)	-	03:05	02:45	03:30	-	03:00	02:00	03:30	-	02:53	01:30	03:25

Note. *Total *N* of meetings observed across all 3 phases = 30. **Total *N* of patients discusses across all 3 phases = 1,335.

For peer review only

Table 2. Results from multiple comparison tests

**Comparison 1 (horizontal, Figure 2): Information scores in 1st versus 2nd half of the meeting**
Mean information scores for 1st and 2nd half of meetings were 16.36±2.49 and 15.10±3.34 in phase 2, and in phase 3 they were 16.00±3.96 and 15.94±3.61, respectively. In phase 2, mean information score was significantly higher in the 1st as opposed to 2nd half of the meeting, 1.26 (95% CI, 0.64 to 1.88), $p<0.001$, and in phase 3 mean difference was non-significant, 0.06 (95% CI, -0.53 to 0.64), $p=0.845$.
**Comparison 2 (vertical, Figure 2): Information scores pre- versus post-break**
Mean information score in phases 2 and 3 did not significantly differ in the 1st half of the meeting, 0.36 (95% CI, -0.24 to 0.96) $p=0.238$; however, in the 2nd half of the meeting, mean information score was significantly higher in phase 3 than phase 2, -0.84 (95% CI -1.45 to -0.24), $p<0.01$.
**Comparison 3 (horizontal, Figure 2): Contribution scores in 1st versus 2nd half of the meeting**
Mean *contribution scores* for 1st and 2nd half of the meeting were 22.67±2.83 and 21.52±3.87 in phase 2, and in phase 3 they were 17.66±5.35 and 19.85±5.43, respectively. In phase 2, mean *contribution score* was significantly higher in the 1st as opposed to the 2nd half of the meeting, 1.15 (95% CI, 0.32 to 1.98), $p<0.01$, and in phase 3, the mean was significantly lower in the 1st as opposed to the 2nd half of the meeting, -2.19 (95% CI, -2.97 to -1.41), $p<0.001$.
**Comparison 4 (vertical, Figure 2): Contribution scores pre- versus post-break**
In the 1st half of the meeting, mean *contribution score* was significantly higher in phase 2 than phase 3, 5.01 (95% CI, 4.21 to 5.81) $p<0.001$; however, in the 2nd half of the meeting, the mean was significantly higher in phase 2 than in phase 3, 1.67 (95% CI 0.86 to 2.48), $p<0.001$.

Table 3. Pearson correlation between ordinal position of cases and the information and contribution scores

	**Information score**	**Contribution score**	***n***
Ordinal position of patients in **phase 1**	-0.254*	-0.160*	346
Ordinal position of patients in **phase 2**	-0.206*	-0.128*	467
Ordinal position of patients in **phase 3**	-0.078	0.072	522

Note. *$p < 0.01$. $N = 1{,}335$ patient-reviews.

peer review only

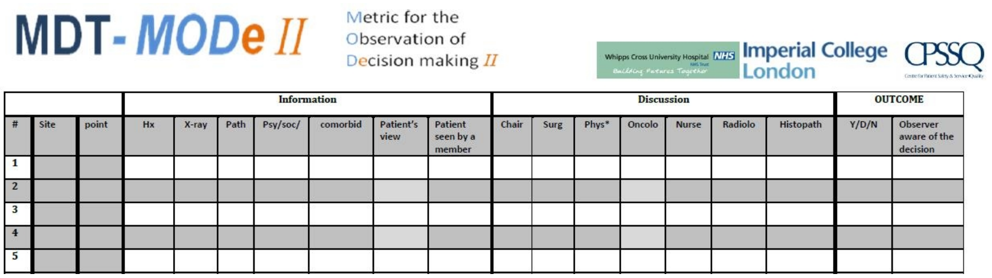

			Information							Discussion							OUTCOME	
#	Site	point	Hx	X-ray	Path	Psy/soc/	comorbid	Patient's view	Patient seen by a member	Chair	Surg	Phys*	Oncolo	Nurse	Radiolo	Histopath	Y/D/N	Observer aware of the decision
1																		
2																		
3																		
4																		
5																		

Figure 1. Metric for the observation of decision-making in cancer multidisciplinary team meetings (MDT-MODe)

218x59mm (144 x 144 DPI)

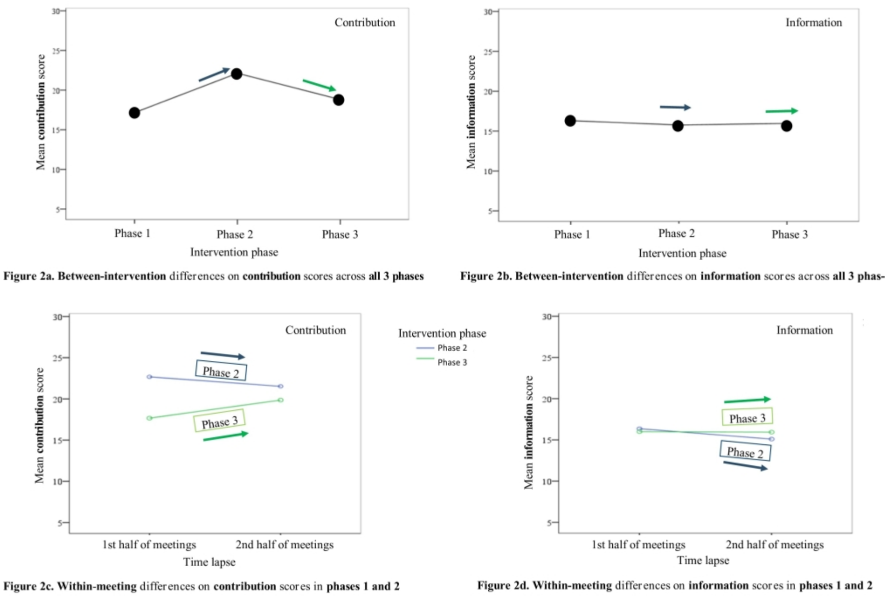

Figure 2. Mean scores for information and contribution quality across the observational phases 1, 2 and 3, as well as across the 1st and 2nd half of the meetings in phases 2 and 3 only

297x209mm (300 x 300 DPI)

STROBE Statement

Checklist of items that should be included in reports of observational studies

Section/Topic	Item No	Recommendation	Reported on Page No
**Title and abstract**	1	(*a*) Indicate the study's design with a commonly used term in the title or the abstract	1
		(*b*) Provide in the abstract an informative and balanced summary of what was done and what was found	3
**Introduction**			
Background/rationale	2	Explain the scientific background and rationale for the investigation being reported	4-5
Objectives	3	State specific objectives, including any prespecified hypotheses	5
**Methods**			
Study design	4	Present key elements of study design early in the paper	5-6
Setting	5	Describe the setting, locations, and relevant dates, including periods of recruitment, exposure, follow-up, and data collection	6
Participants	6	(*a*) *Cohort study*—Give the eligibility criteria, and the sources and methods of selection of participants. Describe methods of follow-up<br>*Case-control study*—Give the eligibility criteria, and the sources and methods of case ascertainment and control selection. Give the rationale for the choice of cases and controls<br>*Cross-sectional study*—Give the eligibility criteria, and the sources and methods of selection of participants	6
		(*b*) *Cohort study*—For matched studies, give matching criteria and number of exposed and unexposed<br>*Case-control study*—For matched studies, give matching criteria and the number of controls per case	
Variables	7	Clearly define all outcomes, exposures, predictors, potential confounders, and effect modifiers. Give diagnostic criteria, if applicable	8
Data sources/measurement	8*	For each variable of interest, give sources of data and details of methods of assessment (measurement). Describe comparability of assessment methods if there is more than one group	7
Bias	9	Describe any efforts to address potential sources of bias	9
Study size	10	Explain how the study size was arrived at	6
Quantitative variables	11	Explain how quantitative variables were handled in the analyses. If applicable, describe which groupings were chosen and why	8
Statistical methods	12	(*a*) Describe all statistical methods, including those used to control for confounding	9
		(*b*) Describe any methods used to examine subgroups and interactions	9
		(*c*) Explain how missing data were addressed	9
		(*d*) *Cohort study*—If applicable, explain how loss to follow-up was addressed<br>*Case-control study*—If applicable, explain how matching of cases and controls was addressed<br>*Cross-sectional study*—If applicable, describe analytical methods taking account of sampling strategy	n/a
		(*e*) Describe any sensitivity analyses	n/a

For peer review only - http://bmjopen.bmj.com/site/about/guidelines.xhtml

Section/Topic	Item No	Recommendation	Reported on Page No
**Results**			
Participants	13*	(a) Report numbers of individuals at each stage of study—eg numbers potentially eligible, examined for eligibility, confirmed eligible, included in the study, completing follow-up, and analysed	9
		(b) Give reasons for non-participation at each stage	n/a
		(c) Consider use of a flow diagram	n/a
Descriptive data	14*	(a) Give characteristics of study participants (eg demographic, clinical, social) and information on exposures and potential confounders	9
		(b) Indicate number of participants with missing data for each variable of interest	n/a
		(c) *Cohort study*—Summarise follow-up time (eg, average and total amount)	n/a
Outcome data	15*	*Cohort study*—Report numbers of outcome events or summary measures over time	
		Case-control study—Report numbers in each exposure category, or summary measures of exposure	
		Cross-sectional study—Report numbers of outcome events or summary measures	10-12
Main results	16	(*a*) Give unadjusted estimates and, if applicable, confounder-adjusted estimates and their precision (eg, 95% confidence interval). Make clear which confounders were adjusted for and why they were included	n/a
		(*b*) Report category boundaries when continuous variables were categorized	n/a
		(*c*) If relevant, consider translating estimates of relative risk into absolute risk for a meaningful time period	n/a
Other analyses	17	Report other analyses done—eg analyses of subgroups and interactions, and sensitivity analyses	10-12
**Discussion**			
Key results	18	Summarise key results with reference to study objectives	12-13
Limitations	19	Discuss limitations of the study, taking into account sources of potential bias or imprecision. Discuss both direction and magnitude of any potential bias	14
Interpretation	20	Give a cautious overall interpretation of results considering objectives, limitations, multiplicity of analyses, results from similar studies, and other relevant evidence	13
Generalisability	21	Discuss the generalisability (external validity) of the study results	14
**Other Information**			
Funding	22	Give the source of funding and the role of the funders for the present study and, if applicable, for the original study on which the present article is based	1

**Give information separately for cases and controls in case-control studies and, if applicable, for exposed and unexposed groups in cohort and cross-sectional studies.*

Note: An Explanation and Elaboration article discusses each checklist item and gives methodological background and published examples of transparent reporting. The STROBE checklist is best used in conjunction with this article (freely available on the Web sites of PLoS Medicine at http://www.plosmedicine.org/, Annals of Internal Medicine at http://www.annals.org/, and Epidemiology at http://www.epidem.com/). Information on the STROBE Initiative is available at www.strobe-statement.org.

For peer review only - http://bmjopen.bmj.com/site/about/guidelines.xhtml

For peer review only

Do multidisciplinary cancer care teams suffer decision-making fatigue? An observational, longitudinal team improvement study

Journal:	*BMJ Open*
Manuscript ID	bmjopen-2018-027303.R1
Article Type:	Research
Date Submitted by the Author:	25-Jan-2019
Complete List of Authors:	Soukup, Tayana ; King's College London, Centre Implementation Science; King's College London<br>Gandamihardja, Tasha; Chelmsford Breast Unit<br>McInerney, Sue; Imperial College Healthcare NHS Trust<br>Green, James; Barts Health NHS Trust, Department of Urology, Whipps Cross University Hospital; London South Bank University<br>Sevdalis, Nick; King's College London,
**Primary Subject Heading**:	Oncology
Secondary Subject Heading:	Oncology
Keywords:	Cancer multidisciplinary team meetings, decision-making, decision-making fatigue

SCHOLARONE™
Manuscripts

Do multidisciplinary cancer care teams suffer decision-making fatigue? An observational, longitudinal team improvement study

Tayana Soukup PhD[a]✉, Tasha Gandamihardja MEd PhD FRCS[b], Sue McInerney RGN[c], James S. A. Green LLM FRCS (Urol)[ad], Nick Sevdalis PhD[a]

Affiliations List

[a] Centre for Implementation Science, King's College London, UK

[b] Chelmsford Breast Unit, Broomfield Hospital, Chelmsford, UK

[c] Imperial College Healthcare NHS Trust, Charring Cross Hospital London, UK

[d] Whipps Cross University Hospital, London, UK

Corresponding author

✉ Dr Tayana Soukup, King's College London, Health Services and Population Department, Demark Hill, London SE5 8AF, E-mail: tayana.soukup@kcl.ac.uk Tel.: +44 20 7848 0683.

Short Title

Decision-making fatigue in oncology meetings

Keywords

Cancer multidisciplinary team meetings; decision-making; decision-making fatigue;

Word count

4424 words

For peer review only

ABSTRACT

Objective

The objective of this study was to examine effectiveness of co-designed quality-improving interventions with a multidisciplinary team (MDT) with high workload and prolonged meetings to ascertain: (1) presence and impact of decision-making (DM) fatigue on team performance in the weekly MDT meeting, and (2) impact of a short meeting break as a countermeasure of DM fatigue.

Design and interventions

This is a longitudinal multiphase study with a co-designed intervention bundle assessed within team audit and feedback cycles. The interventions comprised short meeting breaks, as well as change of room layout and appointing a meeting chair.

Setting and participants

A breast cancer MDT with 15 members was recruited between 2013 and 2015 from a teaching hospital of the London (UK) metropolitan area.

Measures

A validated observational tool (Metric for the Observation of Decision-making, MDT-MODe) was used by trained raters to assess quality of DM during 1,335 patient-reviews. The tool scores quality of information and team contributions to reviews by individual disciplines (Likert-based scores), which represent our two primary outcome measures.

Results

Data were analysed using multivariate analysis of variance. DM fatigue was present in the MDT meetings: quality of information (*M*=16.36 to *M*=15.10) and contribution scores (*M*=27.67 to *M*=21.52) declined from 1st to 2nd half of meetings at baseline. Of the improvement bundle, we found breaks reduced the effect of fatigue: following introduction of breaks (but not other interventions) information quality remained stable between 1st and 2nd half of meetings (*M*=16.00 to *M*=15.94), and contributions to team DM improved overall (*M*=17.66 to *M*=19.85).

Conclusion

Quality of cancer team DM is affected by fatigue due to sequential case-review over often prolonged periods of time. This detrimental effect can be reversed by introducing a break in the

For peer review only

middle of the meeting. The study offers a methodology based on 'team audit and feedback' principle for co-designing interventions to improve teamwork in cancer care.

For peer review only

STRENGTHS

1. A validated tool was used
2. Subset of cases was scored by trained evaluators in pairs blind to one another's scores
3. Main assessor was a clinician whose presence in MDT meetings is natural

LIMITATIONS

1. Observer bias and Hawthorne effect
2. Pre-post study design with no control over extraneous elements that are changing at the same time as the intervention is implemented

For peer review only

INTRODUCTION

In the UK, care planning for patients with cancer is routinely (and mandatorily) carried out by a multidisciplinary team (MDT), generally consists of histopathologists, radiologists, surgeons, specialist cancer nurses and oncologists, in typically weekly meetings (or tumour boards). Here, patients are reviewed and treatment recommendations are agreed upon by the team in a sequential manner for up to a few hours at a time.[1-9] While the MDT approach to cancer care is endorsed widely,[7] evidence of its effectiveness is unclear and variable.[8-17] A pattern generally observed in MDT meetings is unequal participation to discussion and suboptimal sharing of information.[1,8-16] Evidence from studies on small groups suggests that variability in performance is attributable to human factors, such as those that are internal to teams incl. leadership, group composition and personality traits, as well as the external circumstances, such as increasing workload, time pressures, and shifting economic landscape.[18]

Hence one aspect of MDT meetings warrants further focus, and that is the type of fatigue that arises as a result of increasing workload. To-date, evidence has documented high workloads on cancer MDTs with meetings up to 5h reported in the recent Cancer Research UK report.[5] For example, in the UK, studies have reported that a breast cancer MDT reviewed between 29 and 51 patients with the meeting often running for up to 3.5h;[1] lung MDT between 22 and 30 patients with meetings up to 3h;[2] urology MDT between 19 and 51 patients with meetings up to 2h;[3] and a colorectal MDT between 9 and 55 patients with meetings up to 1h and 40min.[4] High workloads and prolonged periods of consecutive DM in the meetings have become a norm for many teams,[6,8] something that is likely to continue as teams are trying to maximise productivity in the face of increasing numbers of new cancer cases worldwide,[19-20] rising financial pressures,[20-21] and growing staff shortages.[22]

Little is known however about the impact of such intense periods of cognitive activity on clinical performance with one study showing that the quality of endoscopy performance declines with repetitive procedures i.e. when conducted one after another for a prolonged period of time.[23] Evidence from cognitive science shows that such consecutive cognitive efforts on a task can lead to cognitive depletion, negatively affecting subsequent decisions, leading to performance decrements over time – also known as *decision-making fatigue (DM fatigue)*.[24] Consequences are many, including: rushed decisions, lack of attention to all available information and potential implications, status quo,[25-26] reduced ability to effectively evaluate choices and sustain attention,

For peer review only

as well as easy distractibility and absentmindedness.[27-29] Strategies, such as short breaks, consuming food, glucose and water can help safeguard against decision fatige,[24,30-35] something that in other industries, such as aviation, has been recognisied.[34-35]

This is not the case for healthcare, however. On the one hand, the World Health Organisation[36] recognises general fatigue as a leading contributor to medical error, and European Working Time Directive[37] restricts excessive night work and working hours. On the other hand, the type of fatigue that arises because of intensity and complexity of workload during working hours has not received the same level of recognition; despite healthcare being fraught with examples of intense cognitive work.[38-40] To-date, the impact of DM fatigue has not been explored in healthcare settings; our objective was to examine this concept for the first time within the current study design.

One way of testing and evaluating the concept of DM fatigue with an MDT is to apply the principles of 'team audit and feedback' - a process of providing non-punitive and actionable feedback to professionals to allow them to self-assess and adjust their performance, thus stimulating desired behaviour change.[41-43] Such approach was found effective in improving practice and supporting quality improvements, and can be used to aid implementation of evidence-based interventions.[41] Within our study, this approach allowed us to elicit inputs from all team members, which we then used to co-design interventions to best meet the needs of the team in addressing DM fatigue. As a team-centred approach to intervention development, implementation and evaluation, this is, to the best of our knowledge, yet to be applied to cancer MDTs.

Aim and objectives

The overarching aim of our study was to identify and co-design quality-improving team interventions (in feedback sessions), and test their effectiveness (in team audits) with an MDT with high workload and prolonged meetings.

Within this overarching aim, we had two specific objectives based on the challenging circumstances the team was in with long meetings and high workload, and the scientific knowledge-base on fatigue that can arise in such challenging circumstances.[23-35] It was therefore reasonable to explore in such concrete setting (1) the presence and impact of DM fatigue on team performance in MDT meetings, and (2) the impact of a short break in MDT meetings as a countermeasure of DM fatigue.

For peer review only - http://bmjopen.bmj.com/site/about/guidelines.xhtml

METHODS

Study design

This was a longitudinal prospective observational study carried out over a 2-year period with a breast cancer MDT. Interventions were introduced within a single arm pre-post study design in order to allow us to identify and co-design interventions (in feedback session), and test whether these interventions work under difficult real-life circumstances where workload is high and meetings exceptionally long (in team audit).

Patient and public involvement

Patients and public were not involved in the development and design of this study.

Setting

A breast cancer MDT was recruited between 2013 and 2015 from a teaching hospital of the London (UK) metropolitan area.

Participants

Participants were 15 members of a breast cancer team, and a total of 1,335 breast cancer patients reviewed at 30 MDT meetings. Availability sampling was used to identify the team with a criterion for the study being a cancer MDT from the UK National Health Service (NHS) that represents one of the most common types of cancer, and experiences high workload with prolonged meeting duration (>1h). Sample size in terms of number of MDT meetings per study phase (n=10) was determined based on our feasibility study,[1] and a prior study of our group in urology with similar workload.[12] The study was granted Ethical Approval by the local ethics committee (JRCO REF. 157441).

Intervention Design: Audit and Feedback Cycles

Interventions were co-designed and evaluated based on the principles of team audit and feedback.[41-42] In what follows, we outline what this process entailed.

Audit cycles focused on collecting observational data of team DM processes across 3 phases. In **phase 1** (baseline; MDT meetings 1 to 10; July to Nov 2013), we did not introduce any interventions, but observations of care as usual. The descriptive data from this phase have been reported as a pilot study to establish feasibility of the measurement.[1] In **phase 2** (MDT meetings 11 to 20; Feb to April 2014), we introduced two interventions including (1) *change of the room*

For peer review only - http://bmjopen.bmj.com/site/about/guidelines.xhtml

layout from lecture theatre style to a U-shape where team-members were able to face each other, and (2) formal appointment of an *MDT meeting chair-person*. The rationale for these interventions was that the change of the room layout will be more conducive to team interactions, while appointment of the formal chair will help facilitate the overall flow of the meeting and individual patient discussions. In the final **phase 3** (MDT meetings 21 to 30; Sept 2014 to March 2015), we introduced a 10-minute long break for tea, coffee and snacks halfway through the MDT meetings, i.e., typically at the 90-minute mark, which was hypothesised to help counteract negative effects of DM fatigue.

Feedback sessions focused on identifying and co-designing interventions. The interventions were identified and chosen based on the observational data from each phase, MDT recommendations, guidelines and evidence-base, as well as on team discussion and consensus within each feedback session. I.e. in each feedback session, the data from previous phase was presented to the team. The data was then benchmarked against previous observational phase, guidelines, recommendations and evidence base for cancer MDTs. In the light of this information, we discussed potential evidence-based interventions that were most appropriate and acceptable to the entire team by reaching a consensus.

More specifically, the feedback sessions occurred at 3 time points at the end of each audit phase – in June 2014, May 2014 and June 2015. Each session was allocated a 1h slot as part of the MDT meeting where we **(a)** fed back the summary of the analysis (20-minutes), **(b)** facilitated team-based review of the findings and what they meant for the team (20-minutes); and **(c)** shortlisted evidence-based interventions the team were willing to introduce into their work in the coming study period (20-minutes).

The process of implementing interventions was agreed upon in the feedback sessions, and it was facilitated/enabled in a collaborative manner. Specifically, following each feedback sessions, the research team produced minutes and actions that were approved and emailed to the MDT by their lead, a Consultant Breast Surgeon (TG). The MDT was invited to comment and identify date for intervention implementation. The task of leading the introduction/implementation of the interventions was assigned to the MDT lead. Interventions were introduced and allowed a 'bed-in' period of approximately 3 months, during which no assessments were carried out to allow the team to familiarise themselves with the novel way of working. This approach was designed at the

For peer review only

For peer review only - http://bmjopen.bmj.com/site/about/guidelines.xhtml

request of the MDT who needed the 'bed-in' time to ensure they did not feel they were being 'examined' by the research team at a time when they were in a state of change. The implementation process was led by the MDT, therefore.

Materials

We used a validated quantitative observational assessment tool, namely the Metric for Observation of Decision-making, (MDT-MODe; Figure 1),[10] which was tested for feasibility in our pilot study.[1] The tool has been used previously to assess various cancer MDT meetings and has shown good validity and reliability (on individual variables and composite scores).[1-4,10-14]

The MDT-MODe captures the following aspects in a meeting:

1) *Quality of presented patient information*, which includes 6 individual variables scored on a behaviourally anchored 5-point scale, namely, patients' case history, radiological images, histopathology, psychosocial issues, co-morbidities and their views on treatment options. The sum of the scores for all 6 variables represents overall quality of presented information for a patient with the higher scores indicating better quality.

2) *Quality of disciplinary contribution to patient-reviews*, which includes 6 individual variables scored on a behaviourally anchored 5-point scale, representing the surgeons, oncologists, radiologists, histopathologists, BCNs and the chair-person. However, there was no formally appointed meeting chair in the participating team, and so this variable was not scored and analyzed. The sum of the scores for all 6 variables represents overall quality of disciplinary contribution for a patient with the higher scores indicating better quality.

Figure 1

Assessor training

Prior to the formal scoring during the study, the evaluator (Cancer Nurse Specialist, SM) was trained in the use of the MDT-MODe.[10] Training is essential to be able to use it, which is a general principle for instruments assessing human factors in clinical environments.[44] Training was delivered by our team and it involved: (1) explanation of the domains, scales and their anchors, (2) background reading of peer-reviewed literature on the tool, and (3) calibration of scoring against an expert evaluator (TS) via scoring a set of pre-recorded MDT videos.

For peer review only - http://bmjopen.bmj.com/site/about/guidelines.xhtml

To ensure reliability in the use of the tool, a cross-section of the data was double-rated blindly by trained clinical (SM) and psychologist (TS) observers. To minimise H*awthorne effect*, i.e., teams changing their usual behavior due to being observed, the main study evaluator was the Cancer Nurse Specialist, the presence of whom within an MDT meeting is natural. During data collection, each evaluator was blind to the other evaluators' observations, and the observer (SM) did not participate in the MDT meetings clinically. Proficiency in scoring was set as an achievement of inter-assessor reliability of 0.70 or higher between the trainee and expert assessor; [44] this was met.

Statistical methods and variables

There were two independent variables (IVs) in the study:

- IV1 was defined as the '*study phase'* with 3 levels (phases 1, 2 and 3) in the one-way multivariate analysis, and 2 levels (phases 2 and 3) in the two-way multivariate analysis;
- IV2 was defined as the '*time lapse'* with 2 levels, namely, 1st and 2nd half of the meeting.

There were two dependent variables (DVs):

- DV1 is *quality of presented patient information* to the team as measured by MDT-MODe,[10,1]
- DV2 is *quality of disciplinary contributions to patient-review* as measured by MDT-MODe[10,1]

Three sets of analyses were conducted:

1. *Intra-class correlation coefficient (ICC)* analysis was used to assess reliability of evaluations in each phase. ICCs can range between 0 and 1, with higher values indicating better agreement.
2. *Multivariate analysis of variance (MANOVA*) was used to assess:
   a. *between-intervention differences* in DM where the effect of co-designed interventions across all 3 phases is explored using a *one-way MANOVA* with *post-hoc tests*;
   b. *within-meeting differences* in DM where presence of DM fatigue and effect of a 10-min break in phases 2 and 3 is explored using *two-way MANOVA* with *simple main effects*.
3. *Correlation analysis* was used to ascertain presence of DM fatigue across all 3 phases.

All analyses were carried out using SPSS® version 20.0. All pairwise comparisons are reported with Bonferroni-adjusted *p*-values.

For peer review only - http://bmjopen.bmj.com/site/about/guidelines.xhtml

RESULTS

Meeting characteristics

The sample consisted of overall 1,335 patients managed across the 3 study phases (see Table 1). All case-reviews for the duration of the study were conducted in the context of the set interventions. It is evident that the total number of patients discussed per phase steadily increased as the study progressed, which suggests increasing workload for the team over time.

Table 1

Reliability of evaluations

Agreement between evaluators was assessed on a randomly selected subset of patient-reviews within each phase. The selection was driven predominantly by the pragmatic considerations and the availability of the second assessor who was not a member of the participating MDT and was blinded to the patient list for the meetings and the first assessor's scores.

We used single measures interclass correlation with the two-way mixed effects model and an absolute agreement definition. High reliability was obtained across all phases:

- *Baseline/Phase 1*: information $r = 0.89$, contribution $r = 0.82$, $n = 116$, 34% of the cohort;
- *Phase 2*: information $r = 0.92$, contribution $r = 0.95$, $n = 116$, 25% of the cohort;
- *Phase 3*: information $r = 0.88$, contribution $r = 0.79$, $n = 131$, 25% of the cohort.

Between-intervention differences in decision-making across all 3 phases

A one-way MANOVA was run on the dataset[45] to address the overarching aim of the study i.e. to examine effectiveness of co-designed interventions across all 3 study phases.

Specifically, a one-way MANOVA was run to determine effect of co-designed interventions (IV1 with 3 levels: phases 1, 2 and 3) on the information (DV1) and contribution (DV2) scores of the MDT-MODe.[10] Data are expressed as mean ± standard deviation. To preserve statistical power, Bonferroni adjusted p-level of 0.025 was used.

Information scores were similar between phase 1, 2 and 3 (16.31±3.71; 15.76±2.98 and 15.97±3.77, respectively), while the *contribution scores* were lower in phase 1 than 2 and 3 (17.16±3.23; 22.13±3.40; 18.81±5.50, respectively). There is statistically significant difference between the intervention phases on the *combined DVs*, $p<0.001$.

For peer review only - http://bmjopen.bmj.com/site/about/guidelines.xhtml

Follow-up univariate ANOVAs showed that the *information scores* alone **did not** reveal significant differences between phases (p=0.09), while the *contribution scores* **did** (p<0.025). Bonferroni post-hoc tests revealed that for *contribution scores*, phase 2 had significantly higher mean score than phases 1 (p<0.02) and 3 (p>0.02); and that phase 3 had significantly higher mean score than phase 1 (p<0.02).

In sum, the findings show that the *quality of information* remained largely similar across phases, while the *quality of contribution* improved in phases 2 and 3 relative to phase 1 but with no linear improvement across phases. See figure 2 (a and b) for a graphical representation of the results.

Figure 2

Within-meeting differences in decision-making in phases 2 and 3

A two-way MANOVA was run on the dataset[45] to address the two objectives in our study i.e. (1) the presence and impact of DM fatigue on team performance in MDT meetings, and (2) the impact of a short break in MDT meetings as a countermeasure of DM fatigue.

Specifically, a two-way MANOVA was conducted to examine interaction effects between *IV1 or a 10-minute break* (two levels: phase 2 meetings with no break, and phase 3 meetings with a break), and IV2 or '*time lapse'* (two levels: 1st and 2nd half of meetings) on the information (DV1) and contribution (DV2) scores of the MDT-MODe.[10] Data are expressed as mean ± standard deviation.

There was a significant interaction effect between *10-minute break* and *time lapse* on the *information* (p<0.01) and *contribution scores* (p<0.001). An *analysis of simple main effects* for a *10-minute break* and *time lapse* was performed with significance Bonferroni-adjusted for p<0.0125.

Mean information scores for 1st and 2nd half of meetings were 16.36±2.49 and 15.10±3.34 in phase 2, and in phase 3 they were 16.00±3.96 and 15.94±3.61, respectively. There was a significant difference in mean *information scores* for 1st v. 2nd half of the meeting in phase 2 (p<0.001) and a non-significant difference in phase 3 when the meeting break was introduced (p=0.845). Mean information score in phases 2 and 3 did not significantly differ in the 1st half of the meeting, 0.36 (95% CI, -0.24 to 0.96) p=0.238; however, in the 2nd half of the meeting, mean information score was significantly higher in phase 3 than phase 2, -0.84 (95% CI -1.45 to -0.24), p<0.01.

For peer review only - http://bmjopen.bmj.com/site/about/guidelines.xhtml

Mean *contribution scores* for 1st and 2nd half of the meeting were 22.67±2.83 and 21.52±3.87 in phase 2, and in phase 3 they were 17.66±5.35 and 19.85±5.43, respectively. There was also a significant difference in mean *contribution scores* for 1st v. 2nd half of the meeting in phase 2 (p<0.01), and also in phase 3 (p<0.001). In phase 2, mean *contribution score* was significantly higher in the 1st as opposed to the 2nd half of the meeting, 1.15 (95% CI, 0.32 to 1.98), p<0.01, and in phase 3, the mean was significantly lower in the 1st as opposed to the 2nd half of the meeting, -2.19 (95% CI, -2.97 to -1.41), p<0.001.

Figure 2 above for a graphical representation of the comparisons reported here.

In sum, *quality of information and contribution* was reduced in the 2nd half of the meeting when the MDT did not have a 10min break (phase 2). In contrast, when the MDT had a break (phase 3), the quality of information remained unchanged, while the quality of contribution improved. See figure 2 (c and d) for a graphical representation of the results.

Correlation analysis: ordinal position of cases and quality of dm across study phases

A follow-up analysis was conducted on the ordinal position of cases within meetings, and information and contribution scores *to ascertain* performance decrements across all 3 phases, and improvements obtained in phase 3 because of a 10-minute break. Ordinal position of a case within an MDT meeting is taken as an indicator of potential effects of DM fatigue: the later a case is reviewed during the MDT meeting, the more cases the team would have reviewed in a sequential manner prior to it.

Table 2 shows significant negative correlations between ordinal position of cases, and contribution and information scores in phases 1 and 2 - i.e., as the ordinal position of cases increases (i.e., the patient is reviewed later in the meeting), the information and contribution scores decrease (i.e., team interaction and clinical input measures worsen). In phase 3, however, when the short break was introduced, both coefficients are non-significant, indicating overall improvement – i.e., a lack of impact of the repetitive DM process on the team interaction and clinical input indicators.

Table 2

Table 2 also shows that the intervention package introduced in phase 2 (change of room layout and appointing a meeting-chair) did not influence the quality of DM when assessed within

For peer review only

meetings; these effects are only detectable in the between-intervention analysis (see Figure 2a and 2b for a graphical representation of these effects).

Team's feedback on the conduct of the meetings

In the final feedback session (June 2015), the team recognised that the meeting break and seating rearrangement were useful and had positive impact on their working, while appointing a rotating chairperson presented with challenges and is something that would need more focus in order to ensure consistency across weekly meetings. The team reported two reasons for this, one, team friction and lack of clarity around who is chairing, and second, fatigue that the chairperson experiences by having to chair the meeting and contribute clinically to discussion ('chairing fatigue'). The team proposed that, going forward, this could be addressed by assigning the chairing role to another member of the team in the 2nd half of the meeting.

Hence while the fidelity of intervention delivery was good throughout – in particular for the meeting break and change of room layout which were implemented as agreed/planned in the feedback sessions, appointing a meeting chair was more challenging as it appears that although a rotating chair was appointed throughout, due to team friction, not all appointed chairs were accepted by other members of the team in the same manner.

DISCUSSION

The overall aim of this study was to examine effectiveness of co-designed interventions with a breast cancer team with high workload and prolonged meeting duration, and within this, explore presence and impact of DM fatigue, and a short break as a countermeasure. Our findings were threefold. Firstly, our study lends support for the concept of DM fatigue in MDT meetings.[23-24] In phase 2, the information and contribution quality were significantly lower in the 2nd v. 1st half of the meeting. The serial position of cases in the meetings in phases 1 and 2 were also negatively correlated with information and contribution quality, indicating performance decrements as meetings progressed. Secondly, our study lends support to a premise that short break in the middle of a meeting can counterbalance the effect of DM fatigue.[24,30-35] For instance, after the break was introduced in phase 3, serial position of cases no longer showed significantly negative correlation with information and contribution quality, and the scores in the 2nd half of the meeting no longer showed significant decrease.

For peer review only - http://bmjopen.bmj.com/site/about/guidelines.xhtml

Thirdly, we found a significant increase in information and contribution quality after the introduction of co-designed interventions in phases 2 and 3 in comparison to baseline (or, phase 1). This somewhat lends support to co-designed interventions via audit and feedback.[41-43] However, a significant decrease was evident in phase 3 in comparison to phase 2, pointing to challenges at sustaining initially implemented interventions over time. In line with the final team's feedback, one explanation may be chairing fatigue and team friction, which highlights the need for continuous quality improvements and implementation science approaches to help improve our understanding of barriers and facilitators to the uptake of evidence-based interventions for cancer MDTs. It is possible that the feedback should be provided to the team at shorter intervals (after every 5th as opposed to every 10th meeting) to help reinforce the agreed change and goals. Another element that could have (also) indirectly contributed to these findings is the steady increase in workload across phases (Table 2), which is known to negatively impact MDT-working.[16-17]

Nonetheless, despite the nonlinear trajectory between phases 2 and 3, the improvements were made in the within-meeting performance i.e. between 1st and 2nd half of the meeting in phase 3 after the 10-minute break was introduced. This lends support to the concept of DM fatigue - i.e., fatigue that arises because of consecutive cognitive efforts in formulating treatment recommendations, previously explored in other fields (e.g. judicial DM).[24-25] Improved quality of discussion between different disciplines is observed when break is introduced with the quality of presented patient information becoming more stable throughout the meeting. What is more, the 10-minute break did not add additional time to the meeting duration (Table 1), indicating that taking a break made the team more time efficient. The concept of DM fatigue has not yet been explored within cancer MDT meetings, and to our knowledge this is the first study of its kind, with implications for the way meetings are currently structured.

Implications

The implications for meeting structure are far-reaching. It is not only the number of hours worked in a 24-hour period, but also the number of consecutive hours, including the type, intensity and complexity of a task, a clinician engages in without adequate break that requires more focus and recognition. Healthcare is a highly demanding work setting, and apart from MDT meetings, there are many examples of cognitively intense settings, including for e.g., ward rounds and intensive care units.[38-39] While the general health worker fatigue is addressed by the European Working Time Directive[37] which restricts excessive night work and working hours, the type of fatigue that

For peer review only - http://bmjopen.bmj.com/site/about/guidelines.xhtml

arises as a result of intensity and complexity of the workload during the working hours is not adequately acknowledged or safeguarded with recommendations, such as a short break, for instance. It is understood however that the fatigue is a leading contributor to medical error and injury,[36] and that intense episodes of workload in healthcare are on the increase,[19-21,5] as clinical teams are trying to maximise productivity in the face of severe staff shortages[22] and financial pressures.[20-21]

Limitations

Our findings need to be interpreted within certain limitations (some of which have been previously reported).[1]

First, participants in our study were aware that they were being observed. This was necessary due to (a) the methodological approach undertaken in our study, i.e. team audit and feedback that requires the results to be fed back to the team and interventions co-designed thus making the research useful to the team, as well as (b) the ethical and regulatory constraints which meant that we had to provide full description of the study to the participants – this is due to the importance of informed consent (in line with the Good Clinical Practice), and the absence of such consent i.e. deception (e.g. where MDT members are not aware that they are being observed) being regarded as high-risk to participants, requiring checks and considerations by the research ethics committee that reviewed current study (where MDT members knew that they were being observed; under JRCO REF. 157441). Hence, we cannot rule out *Hawthorne effect* and the observer bias. While the former is a natural limitation to observational studies, we ensured that the main study evaluator was a clinician, in our case, Cancer Nurse Specialist, the presence of whom within an MDT meeting is natural. In terms of the latter, we used a validated tool with a subset of cases scored by *trained* evaluators in pairs who were blind to one another's observations within each phase of the study.

Second, while this is a large-scale study for its nature (observations in real-time), we acknowledge that there are cancer MDT meetings that are not as long as the ones reported here, hence the *generalizability of our findings may be limited* to MDTs with high workloads and prolonged meeting duration within the NHS setting. However, the global economic and healthcare landscape is rapidly changing – i.e. cancer incidence[19-20] is on the increase, as well as MDT workload,[5,19] financial pressures,[20-21] and staff shortages.[22] The findings that we report may therefore become

For peer review only - http://bmjopen.bmj.com/site/about/guidelines.xhtml

increasingly relevant to MDTs across different tumor types (and other healthcare settings) globally and could be profitably explored to determine the extent to which they apply to them.

Third, our study is of *pre-post design*, which can limit generalizability of our findings. This is because there is no control over other (extraneous) elements that are also changing at the same time as the intervention is implemented. While we understand that randomized controlled trials provide increased control of such extraneous factors allowing better precision in testing the efficacy of interventions, the aim of our study was to examine the effectiveness of interventions that were identified and co-designed with the participating team under the challenging real-world circumstances where workload and meeting duration are exceptionally high. Nonetheless, future research could adopt an RCT approach to testing the co-designed interventions identified as part of our study with multiple different MDTs to ascertain the impact of each on team functioning under ideal controlled circumstances, which in combination with our effectiveness findings with a single team under real-life circumstances would greatly enhance generalisability. However, MDTs tend to have rather different problems and priorities,[46] and so if they opt for a co-designed approach, they may end up with different interventions. Hence one would need to start off with a few smaller scale studies, such is the current one, followed by a wider consensus exercise across MDTs where a selection of team and functional improvement interventions could be identified and prioritised – these could then be designed into a randomised controlled trial.

The strength of our methodological approach resides in a large sample size (*N*=1335), a robust methodology with validated tools and training, and an approach to improvement that is highly team-centred/driven, engaging, inclusive, non-intrusive and feasible for the team (i.e. does not add to their workload). Such approach has allowed us to capture complex organisational behaviour of the MDT in real time, providing good external validity, evidence of effectiveness, while identifying a set of acceptable co-designed interventions for MDTs with high workload and increased meeting duration.

Fourth, the validated tool used in the current study (MDT-MODe) *does not allow for individual person-level assessment*; only disciplinary group-level with the unit of analysis being a case-discussion (and not an individual team member; Figure 1). Such approach has advantages when evaluating a relatively small (single) team because it ensures team safety by minimizing the risk of defensive routine and blaming a particular team member for performance difficulties which could in turn distract the team from addressing their performance problems constructively.[47] We

For peer review only - http://bmjopen.bmj.com/site/about/guidelines.xhtml

acknowledge however that such approach also has limitations because it does not capture (the effect of) team interaction, as well as (the effect of) individual team member's level of seniority, experience, and personality, and so the effect of the physician versus the team, or style of presentation of different radiologists/histopathologists cannot be accounted for. To address these questions, a different methodological approach may be better suited, such as Conversation Analysis for instance, which allows for an in-depth analyses of team interaction on an individual person-level. Also, development of tools for MDTs should take this limitation into account.

Lastly, while the current study is focused on DM process at the point of the MDT meeting, we have not linked these processes to clinical, patient-related outcomes. As a result, the safety implications of this analysis remain exploratory and are not yet equated to clinical outcomes.

Further research

The objective of our study was to investigates presence and impact of fatigue on *DM processes* in a team with high workload; as such, we did not address how it impacts the *quality of decisions reached* (e.g., their clinical suitability for the patient) or *patient outcomes*. This is however an important next step that should be further explored in the light of our findings and previous research showing that DM fatigue leads to impulsive decisions, status quo, and reduced ability to effectively evaluate information – these could potentially have a knock-on effect on patient outcomes.[17-22] Further research is also needed to assess presence of DM fatigue across different cancer MDTs, particularly those with high workloads, and explore effectiveness of various evidence-based cognitive strategies.[24,30-33] Efforts should be channelled toward safeguarding optimal DM in MDT meetings, taking into account the intensity and complexity of the workload, with strategies in place as standard practice - such as, for instance, a maximum limit of cases allowed for a single meeting, mandatory short break (as practiced in the aviation industry), and trained team lead/chairperson to help the team effectively navigate through workload.[6] Team-centred, co-designed approaches may prove useful in helping identify appropriate (tailored) strategies for a team, however, challenges exist at sustaining change over time; hence, a need for continuous quality improvement and implementation science approaches in the field of cancer MDTs.

CONCLUSIONS

Previous research has shown variability in the quality of DM across cancer MDT meetings, with internal factors, such as group composition and leadership, and external circumstances, such as

For peer review only - http://bmjopen.bmj.com/site/about/guidelines.xhtml

increased workload, time pressures and changing economic landscape held accountable. Our study demonstrates for the first time that quality of DM in cancer MDT meetings grows worse during consecutive cognitive efforts and is positively influenced with a break. Using principles of team audit and feedback to co-design team-centred interventions is a useful approach in helping initiate improvements, however, challenges exist at sustaining interventions over time. Building on our findings, further research in MDTs is needed to investigate effects of DM fatigue on the quality of decisions reached and patient outcomes, ascertain its presence across different cancer teams, and encourage implementation of quality-improving strategies to protect optimal DM. The work could be extrapolated to other areas of clinical (and non-clinical) practice and may have implications for other areas that have equally intense periods of cognitively demanding work.

ACKNOWLEDGEMENTS

The authors would like to thank the breast cancer MDT and their members for their time and commitment to this project.

For peer review only

DISCLOSURE

Nick Sevdalis is the Director of London Safety & Training Solutions Ltd, which provides team working, patient safety and improvement skills training and advice on a consultancy basis to hospitals and training programs in the UK and internationally. James Green is a Director of Green Cross Medical Ltd that developed MDT FIT for use by National Health Service Cancer Teams in the UK. The other authors have no conflicts of interest to report.

FUNDING

Financial support for this study was provided entirely by the UK's National Institute for Health Research (NIHR) via the Imperial Patient Safety Translational Research Centre. Sevdalis and Soukup's research is funded by the NIHR via the 'Collaboration for Leadership in Applied Health Research and Care South London' at King's College Hospital NHS Foundation Trust, London, UK. Sevdalis is also a member of King's Improvement Science, which is part of the NIHR CLAHRC South London and comprises a specialist team of improvement scientists and senior researchers based at King's College London. Its work is funded by King's Health Partners (Guy's and St Thomas' NHS Foundation Trust, King's College Hospital NHS Foundation Trust, King's College London and South London and Maudsley NHS Foundation Trust), Guy's and St Thomas' Charity, the Maudsley Charity and the Health Foundation. The funding agreement ensured the authors' independence in designing the study, interpreting the data, writing, and publishing the report. The views expressed are those of the authors and not necessarily those of the NHS, the NIHR or the Department of Health and Social Care.

AUTHORS' CONTRIBUTIONS

In line with the guidelines by the International Committee of Medical Journal Editors, all authors for this study (i.e., TS, TG, SM, JG, and NS) have made substantial contributions to conception and design, or acquisition of data, or analysis and interpretation of data; have been involved in drafting the manuscript or revising it critically for important intellectual content; have given final approval of the version to be published; and have agreed to be accountable for all aspects of the work in ensuring that questions related to the accuracy or integrity of any part of the work are appropriately investigated and resolved.

For peer review only

DATA SHARING STATEMENT

The anonymised dataset supporting this study is available on Zenodo, a research data repository, under the Creative Commons Attribution Non-Commercial Non-Derivative 4.0 license. The researchers are free to reuse and redistribute the data set on the condition that they *attribute it*, that they *do not use it for commercial purposes*, and that they *do not alter it*. For any reuse or redistribution, researchers must make clear to others *the license terms* of this work and cite the dataset accordingly.

For peer review only

REFERENCES

1 Gandamihardja TAK, Soukup T, McInerney S, Green JSA, Sevdalis N. (in press) Analyzing breast cancer multidisciplinary patient management: a prospective observational evaluation of team clinical decision making. *World J Surg* 2018;in press.

2 Sarkar S, Arora S, Soukup T, Lamb BW, Shah S, Green JSA, Sevdalis N, Darzi, A. A multi-centre study evaluating performance of multidisciplinary teams: Urology vs the top cancer killers. *Eur Urol Suppl* 2014;13: e878-e878a.

3 Lamb BW, Sevdalis N, Benn J, Vincent C, Green JS. Multidisciplinary cancer team meeting structure and treatment decisions: A prospective correlational study. *Ann Surg Onc* 2013;20: 715–722.

4 Jalil R, Akhter W, Lamb BW, Taylor C, Harris J, Green JSA. Validation of team performance assessment of multidisciplinary tumor boards. *J Urol* 2014;192(3): 891-898.

5 Cancer Research UK. Improving the effectiveness of multidisciplinary team meetings in cancer services. London, Cancer Research UK; 2017.

6 National Cancer Action Team. The characteristics of an effective multidisciplinary team (MDT). London, UK: National Cancer Action Team; 2010.

7 Department of Health. Manual for Cancer Services. London, UK: The Department of Health; 2004.

8 Raine R, Xanthopoulou P, Wallace I. Determinants of treatment plan implementation in multidisciplinary team meetings for patients with chronic diseases: a mixed-methods study. *BMJ Qual Saf* 2014;23: 867–876.

9 Hong NJ, Wright FC, Gagliardi AR, Paszat LF. Examining the potential relationship between multidisciplinary cancer care and patient survival: An international literature review. *J Surg Oncol* 2010;102: 125-134.

10 Lamb BW, Wong HWL, Vincent C, Green JSA, Sevdalis N. Teamwork and team performance in multidisciplinary cancer teams: Development of an observational assessment tool. *BMJ Qual Saf* 2011;20: 849-856.

11 Lamb BW, Brown K, Nagpal K, Vincent C, Green JS, Sevdalis N. Quality of care management decisions by multidisciplinary cancer teams: A systematic review. *Ann Surg Oncol* 2011;18: 2116-2125.

For peer review only

12 Lamb BW, Green JS, Benn J, Brown KF, Vincent C, Sevdalis N. Improving decision making in multidisciplinary tumor boards: Prospective longitudinal evaluation of a multicomponent intervention for 1,421 patients. *J Am Coll Surg* 2013;217(3): 412-420.

13 Soukup T, Lamb BW, Sarkar S, Arora S, Shah S, Darzi A, Green JSA, Sevdalis N. Predictors of treatment decision in multidisciplinary oncology meetings: A quantitative observational study. *Ann Surg Oncol* 2016;23(13): 4410-4417.

14 Soukup T, Petrides KV, Lamb BW, Sarkar S, Arora S, Shah S, Darzi A, Green JSA, Sevdalis N. The anatomy of clinical decision-making in multidisciplinary cancer meetings: A cross-sectional observational study of teams in a natural context. *Medicine* 2016;95;e3885.

15 Stairmands J., Signal L, Sarfati D, Jackson C, Batten L, Holdaway M, Cunningham C. Consideration of comorbidity in treatment decision-making in multidisciplinary team meetings: A systematic review. *Ann Oncol* 2015;26(7): 1325-1332.

16 Kidger J, Murdoch J, Donovan JL, Blazeby JM. Clinical decision-making in a multidisciplinary gynaecological cancer team: a qualitative study. *BJOG* 2009;116(4): 511-517.

17 Haward R, Amir Z, Borrill C, Dawson J, Scully J, West M, et al. Breast cancer teams: The impact of constitution, new cancer workload, and methods of operation on their effectiveness. *Br J Cancer* 2003;89(1): 15-22.

18 Poole MS, Hollingshead AB. Theories of small groups: Interdisciplinary perspectives. Thousand Oaks, CA: Sage; 2005.

19 Mistry M, Parkin DM, Ahmad AS, Sasieni P. Cancer Incidence in the UK: Projections to the Year 2030. *Br J Cancer* 2011;105: 1795-1803.

20 World Health Organization. World Cancer Report 2014. France: International Agency for Research on Cancer, World Health Organization; 2014.

21 NHS England. Everyone Counts: Planning for Patients 2014/2015 to 2018/2019. England, UK: NHS England; 2014.

22 NHS Improvement. Evidence from NHS Improvement on clinical staff shortages: A workforce analysis. London, NHS Improvement; 2016.

23 Harewood GC, Chrysostomou K, Himy N, Leong WL. Impact of operator fatigue on endoscopy performance: Implications for procedure scheduling. *Dig Dis Sci* 2009;54(8): 1656–1661.

For peer review only

For peer review only - http://bmjopen.bmj.com/site/about/guidelines.xhtml

24 Danziger S, Levav J, Avnaim-Pesso L. (2012). Extraneous factors in judicial decisions. *Proc Natl Acad Sci* 2012;108(17): 6889-6892.

25 Kool W, McGuire JT, Rosen ZB, Botvinick MM. Decision making and the avoidance of cognitive demand. *J Exp Psychol* 2010;139(4): 665-682.

26 Eidelma S, Crandall CS. Bias in favour of the Status Quo. *Soc Personal Psychol Compass* 2012;6(3): 270-281.

27 Weissman DH, Roberts KC, Visscher KM, Woldorff MG. The neural bases of momentary lapses in attention. *Nat Rev Neurosci* 2006;9: 971-978.

28 Kaplan S, Berman MG. Directed attention as a common resource for executive functioning and self-regulation. *Perspect Psychol Sci* 2010;5: 43-57.

29 Sawin DA, Scerbo MW. Effects of instruction type and boredom proneness in vigilance: Implications for boredom and workload. *Hum Factors* 1995;37(4): 752-765.

30 Tyler JM, Burns KC. After depletion: The replenishment of the self's regulatory resources. *Self Identity* 2008;7: 305–321.

31 McMahon AJ, Scheel MH. Glucose promotes controlled processing: Matching, maximising and root beer. *Judgm Decis Mak* 2010;5(6): 450-457.

32 Kempton MJ, Ettinger U, Foster R, Williams SC, Calvert GA, Hampshire A, et al. Dehydration affects brain structure and function in healthy adolescents. *Hum Brain Mapp* 2011;32(1): 71-9.

33 Hagger MS, Chatzisarantis NL. The sweet taste of success: the presence of glucose in the oral cavity moderates the depletion of self-control resources. *Pers Soc Psych Bull* 2013;39: 28–42.

34 Neri DF, Shappell SA, DeJohn CA. Simulated sustained flight operations and performance, Part 1: Effects of fatigue. *Mil Psychol* 1992;4(3): 137-155.

35 Neri DF, Oyung RL, Colletti LM, Mallis MM, Tam PY, Dinges DF. Controlled breaks as a fatigue countermeasure on the flight deck. *Aviat Space Environ Med* 2002;73(7): 654-664.

36 World Health Organisation. WHO Patient Safety Curriculum Guide for Medical Schools. France, A World Alliance for Safer Health Care; 2009.

For peer review only

For peer review only - http://bmjopen.bmj.com/site/about/guidelines.xhtml

37 The European Parliament and the Council of the European Union. Directive 2003/88/EC of the European Parliament and of the Council of 4 November 2003 concerning certain aspects of the organisation of working time. *Official Journal of the European Union*; 2013.

38 Reader TW, Cuthberson BH, Decruyenaere J. Burnout in the ICU: Potential consequences for staff and patient well-being. *Intensive Care Med* 2008;34(1): 4-6.

39 Reader TW, Reddy G, Brett SJ. Impossible decision? An investigation of risk trade-offs in the intensive care unit. *Ergonomics* 2017;61: 122-133.

40 Reid PP, Compton D, Grossman JH, Fanjiang G. Building a better delivery system: A new engineering/health care partnership. Washington, D.C.: The National Academies Press; 2005.

41 Ivers N, Jamtvedt G, Flottorp S, Young JM, Odgaard-Jensen J, French SD. Audit and feedback: effects on professional practice and health care outcomes, Cochrane Database Sys Rev, no. 2, CD000259; 2006.

42 Flottorp SA, Jamtvedt G, Gibis B, McKee M. Using audit and feedback to health professionals to improve the quality and safety of health care. Denmark: World Health Organization on the behalf of the European Observatory on Health Systems and Policies; 2010.

43 Michie S, Johnston M, Abraham C, Lawton R, Parker D, Walker A. Making psychological theory useful for implementing evidence-based practice: a consensus approach. *Qual Saf Health Care* 2005;14: 26-33.

44 Hull L, Arora S, Symons NR, Jalil R, Darzi A, Vincent C. Delphi expert consensus panel. Training faculty in nontechnical skill assessment: national guidelines on program requirements. *Ann Surg* 2013;258(2): 370-375.

45 Soukup, T. Longitudinal intervention in a breast cancer team [Data set]. Zenodo 2018; http://doi.org/10.5281/zenodo.582198

46 Soukup T, Lamb BW, Arora S, Darzi A, Sevdalis N, Green JSA. Successful strategies in implementing a multidisciplinary team working in the care of patients with cancer: An overview and synthesis of the available literature. *J Multidisc Healthc 2018;*11:49-61.

47 West MA. Effective teamwork: practical lessons from organisational research. John Wiley & Sons; 2012.

For peer review only

For peer review only - http://bmjopen.bmj.com/site/about/guidelines.xhtml

FIGURE CAPTIONS

Figure 1. Metric for the observation of decision-making in cancer multidisciplinary team meetings (MDT-MODe)

Figure 2. Mean scores for information and contribution quality across the observational phases 1, 2 and 3, as well as across the 1st and 2nd half of the meetings in phases 2 and 3 only

For peer review only

TABLES

For peer review only

Table 1. Meeting characteristics of the breast cancer team across the intervention phases

**Meeting characteristics**	**Phase 1**				**Phase 2**				**Phase 3**			
	Total	Mean	Min	Max	Total	Mean	Min	Max	Total	Mean	Min	Max
Number of meetings observed*	10	-	-	-	10	-	-	-	10	-	-	-
Number of patients per meeting**	346	42	29	51	467	55	44	73	522	62	52	70
Time per patient-review (MM:SS)	-	03:20	00:31	09:00	-	03:00	00:47	09:06	-	02:06	00:10	12:49
Meeting duration (HH:MM)	-	03:05	02:45	03:30	-	03:00	02:00	03:30	-	02:53	01:30	03:25

Note. *Total *N* of meetings observed across all 3 phases = 30. **Total *N* of patients discusses across all 3 phases = 1,335.

For peer review only

Table 2. Pearson correlation between ordinal position of cases and the information and contribution scores

	Information score	Contribution score	*n*
Ordinal position of patients in **phase 1**	-0.254*	-0.160*	346
Ordinal position of patients in **phase 2**	-0.206*	-0.128*	467
Ordinal position of patients in **phase 3**	-0.078	0.072	522

Note. *$p < 0.01$. $N = 1{,}335$ patient-reviews.

For peer review only

For peer review only - http://bmjopen.bmj.com/site/about/guidelines.xhtml

MDT- MODe II Metric for the Observation of Decision making II

			Information							Discussion							OUTCOME	
#	Site	point	Hx	X-ray	Path	Psy/soc/	comorbid	Patient's view	Patient seen by a member	Chair	Surg	Phys*	Oncolo	Nurse	Radiolo	Histopath	Y/D/N	Observer aware of the decision
1																		
2																		
3																		
4																		
5																		

Figure 1. Metric for the observation of decision-making in cancer multidisciplinary team meetings (MDT-MODe)

218x59mm (144 x 144 DPI)

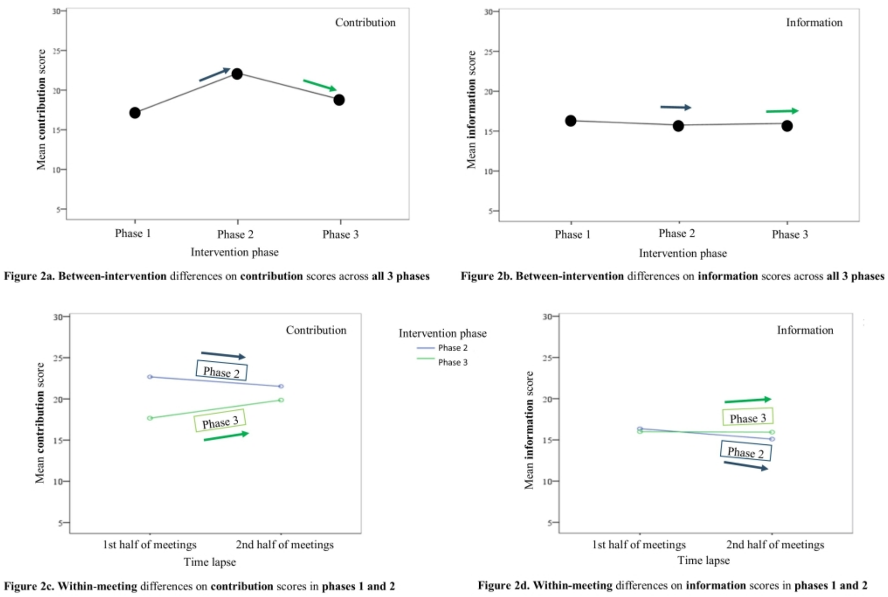

Figure 2. Mean scores for information and contribution quality across the observational phases 1, 2 and 3, as well as across the 1st and 2nd half of the meetings in phases 2 and 3 only

297x209mm (300 x 300 DPI)

STROBE Statement

Checklist of items that should be included in reports of observational studies

Section/Topic	Item No	Recommendation	Reported on Page No
**Title and abstract**	1	(*a*) Indicate the study's design with a commonly used term in the title or the abstract	1
		(*b*) Provide in the abstract an informative and balanced summary of what was done and what was found	3
**Introduction**			
Background/rationale	2	Explain the scientific background and rationale for the investigation being reported	4-5
Objectives	3	State specific objectives, including any prespecified hypotheses	5
**Methods**			
Study design	4	Present key elements of study design early in the paper	5-6
Setting	5	Describe the setting, locations, and relevant dates, including periods of recruitment, exposure, follow-up, and data collection	6
Participants	6	(*a*) *Cohort study*—Give the eligibility criteria, and the sources and methods of selection of participants. Describe methods of follow-up<br>*Case-control study*—Give the eligibility criteria, and the sources and methods of case ascertainment and control selection. Give the rationale for the choice of cases and controls<br>*Cross-sectional study*—Give the eligibility criteria, and the sources and methods of selection of participants	6
		(*b*) *Cohort study*—For matched studies, give matching criteria and number of exposed and unexposed<br>*Case-control study*—For matched studies, give matching criteria and the number of controls per case	
Variables	7	Clearly define all outcomes, exposures, predictors, potential confounders, and effect modifiers. Give diagnostic criteria, if applicable	8
Data sources/measurement	8*	For each variable of interest, give sources of data and details of methods of assessment (measurement). Describe comparability of assessment methods if there is more than one group	7
Bias	9	Describe any efforts to address potential sources of bias	9
Study size	10	Explain how the study size was arrived at	6
Quantitative variables	11	Explain how quantitative variables were handled in the analyses. If applicable, describe which groupings were chosen and why	8
Statistical methods	12	(*a*) Describe all statistical methods, including those used to control for confounding	9
		(*b*) Describe any methods used to examine subgroups and interactions	9
		(*c*) Explain how missing data were addressed	9
		(*d*) *Cohort study*—If applicable, explain how loss to follow-up was addressed<br>*Case-control study*—If applicable, explain how matching of cases and controls was addressed<br>*Cross-sectional study*—If applicable, describe analytical methods taking account of sampling strategy	n/a
		(*e*) Describe any sensitivity analyses	n/a

For peer review only - http://bmjopen.bmj.com/site/about/guidelines.xhtml

Section/Topic	Item No	Recommendation	Reported on Page No
**Results**			
Participants	13*	(a) Report numbers of individuals at each stage of study—eg numbers potentially eligible, examined for eligibility, confirmed eligible, included in the study, completing follow-up, and analysed	9
		(b) Give reasons for non-participation at each stage	n/a
		(c) Consider use of a flow diagram	n/a
Descriptive data	14*	(a) Give characteristics of study participants (eg demographic, clinical, social) and information on exposures and potential confounders	9
		(b) Indicate number of participants with missing data for each variable of interest	n/a
		(c) *Cohort study*—Summarise follow-up time (eg, average and total amount)	n/a
Outcome data	15*	*Cohort study*—Report numbers of outcome events or summary measures over time	
		Case-control study—Report numbers in each exposure category, or summary measures of exposure	
		Cross-sectional study—Report numbers of outcome events or summary measures	10-12
Main results	16	(*a*) Give unadjusted estimates and, if applicable, confounder-adjusted estimates and their precision (eg, 95% confidence interval). Make clear which confounders were adjusted for and why they were included	n/a
		(*b*) Report category boundaries when continuous variables were categorized	n/a
		(*c*) If relevant, consider translating estimates of relative risk into absolute risk for a meaningful time period	n/a
Other analyses	17	Report other analyses done—eg analyses of subgroups and interactions, and sensitivity analyses	10-12
**Discussion**			
Key results	18	Summarise key results with reference to study objectives	12-13
Limitations	19	Discuss limitations of the study, taking into account sources of potential bias or imprecision. Discuss both direction and magnitude of any potential bias	14
Interpretation	20	Give a cautious overall interpretation of results considering objectives, limitations, multiplicity of analyses, results from similar studies, and other relevant evidence	13
Generalisability	21	Discuss the generalisability (external validity) of the study results	14
**Other Information**			
Funding	22	Give the source of funding and the role of the funders for the present study and, if applicable, for the original study on which the present article is based	1

**Give information separately for cases and controls in case-control studies and, if applicable, for exposed and unexposed groups in cohort and cross-sectional studies.*

Note: An Explanation and Elaboration article discusses each checklist item and gives methodological background and published examples of transparent reporting. The STROBE checklist is best used in conjunction with this article (freely available on the Web sites of PLoS Medicine at http://www.plosmedicine.org/, Annals of Internal Medicine at http://www.annals.org/, and Epidemiology at http://www.epidem.com/). Information on the STROBE Initiative is available at www.strobe-statement.org.

For peer review only - http://bmjopen.bmj.com/site/about/guidelines.xhtml

BMJ Open

Do multidisciplinary cancer care teams suffer decision-making fatigue? An observational, longitudinal team improvement study

Journal:	*BMJ Open*
Manuscript ID	bmjopen-2018-027303.R2
Article Type:	Research
Date Submitted by the Author:	22-Mar-2019
Complete List of Authors:	Soukup, Tayana ; King's College London, Centre Implementation Science; King's College London<br>Gandamihardja, Tasha; Chelmsford Breast Unit<br>McInerney, Sue; Imperial College Healthcare NHS Trust<br>Green, James; Barts Health NHS Trust, Department of Urology, Whipps Cross University Hospital; London South Bank University<br>Sevdalis, Nick; King's College London,
**Primary Subject Heading**:	Oncology
Secondary Subject Heading:	Oncology
Keywords:	Cancer multidisciplinary team meetings, decision-making, decision-making fatigue

SCHOLARONE™ Manuscripts

Do multidisciplinary cancer care teams suffer decision-making fatigue? An observational, longitudinal team improvement study

Tayana Soukup PhD[a]✉, Tasha Gandamihardja MEd PhD FRCS[b], Sue McInerney RGN[c], James S. A. Green LLM FRCS (Urol)[ad], Nick Sevdalis PhD[a]

Affiliations List

[a] Centre for Implementation Science, King's College London, UK

[b] Chelmsford Breast Unit, Broomfield Hospital, Chelmsford, UK

[c] Imperial College Healthcare NHS Trust, Charring Cross Hospital London, UK

[d] Whipps Cross University Hospital, London, UK

Corresponding author

✉ Dr Tayana Soukup, King's College London, Health Services and Population Department, Centre for Implementation Science, Demark Hill, London SE5 8AF, E-mail: tayana.soukup@kcl.ac.uk Tel.: +44 20 7848 0683.

Short Title

Decision-making fatigue in oncology meetings

Keywords

Cancer multidisciplinary team meetings; decision-making; decision-making fatigue;

Word count

4424 words

For peer review only

ABSTRACT

Objective

The objective of this study was to examine effectiveness of co-designed quality-improving interventions with a multidisciplinary team (MDT) with high workload and prolonged meetings to ascertain: (1) presence and impact of decision-making (DM) fatigue on team performance in the weekly MDT meeting, and (2) impact of a short meeting break as a countermeasure of DM fatigue.

Design and interventions

This is a longitudinal multiphase study with a co-designed intervention bundle assessed within team audit and feedback cycles. The interventions comprised short meeting breaks, as well as change of room layout and appointing a meeting chair.

Setting and participants

A breast cancer MDT with 15 members was recruited between 2013 and 2015 from a teaching hospital of the London (UK) metropolitan area.

Measures

A validated observational tool (Metric for the Observation of Decision-making, MDT-MODe) was used by trained raters to assess quality of DM during 1,335 patient-reviews. The tool scores quality of information and team contributions to reviews by individual disciplines (Likert-based scores), which represent our two primary outcome measures.

Results

Data were analysed using multivariate analysis of variance. DM fatigue was present in the MDT meetings: quality of information (M=16.36 to M=15.10) and contribution scores (M=27.67 to M=21.52) declined from 1st to 2nd half of meetings at baseline. Of the improvement bundle, we found breaks reduced the effect of fatigue: following introduction of breaks (but not other interventions) information quality remained stable between 1st and 2nd half of meetings (M=16.00 to M=15.94), and contributions to team DM improved overall (M=17.66 to M=19.85).

Conclusion

Quality of cancer team DM is affected by fatigue due to sequential case-review over often prolonged periods of time. This detrimental effect can be reversed by introducing a break in the

For peer review only

middle of the meeting. The study offers a methodology based on 'team audit and feedback' principle for co-designing interventions to improve teamwork in cancer care.

For peer review only

STRENGTHS

1. A validated tool was used
2. Subset of cases was scored by trained evaluators in pairs blind to one another's scores
3. Main assessor was a clinician whose presence in MDT meetings is natural

LIMITATIONS

1. Observer bias and Hawthorne effect
2. Pre-post study design with no control over extraneous elements that are changing at the same time as the intervention is implemented

For peer review only

INTRODUCTION

In the UK, care planning for patients with cancer is routinely (and mandatorily) carried out by a multidisciplinary team (MDT), generally consists of histopathologists, radiologists, surgeons, specialist cancer nurses and oncologists, in typically weekly meetings (or tumour boards). Here, patients are reviewed and treatment recommendations are agreed upon by the team in a sequential manner for up to a few hours at a time.[1-9] While the MDT approach to cancer care is endorsed widely,[7] evidence of its effectiveness is unclear and variable.[8-17] A pattern generally observed in MDT meetings is unequal participation to discussion and suboptimal sharing of information.[1,8-16] Evidence from studies on small groups suggests that variability in performance is attributable to human factors, such as those that are internal to teams incl. leadership, group composition and personality traits, as well as the external circumstances, such as increasing workload, time pressures, and shifting economic landscape.[18]

Hence one aspect of MDT meetings warrants further focus, and that is the type of fatigue that arises as a result of increasing workload. To-date, evidence has documented high workloads on cancer MDTs with meetings up to 5h reported in the recent Cancer Research UK report.[5] For example, in the UK, studies have reported that a breast cancer MDT reviewed between 29 and 51 patients with the meeting often running for up to 3.5h;[1] lung MDT between 22 and 30 patients with meetings up to 3h;[2] urology MDT between 19 and 51 patients with meetings up to 2h;[3] and a colorectal MDT between 9 and 55 patients with meetings up to 1h and 40min.[4] High workloads and prolonged periods of consecutive DM in the meetings have become a norm for many teams,[6,8] something that is likely to continue as teams are trying to maximise productivity in the face of increasing numbers of new cancer cases worldwide,[19-20] rising financial pressures,[20-21] and growing staff shortages.[22]

Little is known however about the impact of such intense periods of cognitive activity on clinical performance with one study showing that the quality of endoscopy performance declines with repetitive procedures i.e. when conducted one after another for a prolonged period of time.[23] Evidence from cognitive science shows that such consecutive cognitive efforts on a task can lead to cognitive depletion, negatively affecting subsequent decisions, leading to performance decrements over time – also known as *decision-making fatigue (DM fatigue)*.[24] Consequences are many, including: rushed decisions, lack of attention to all available information and potential implications, status quo,[25-26] reduced ability to effectively evaluate choices and sustain attention,

as well as easy distractibility and absentmindedness.[27-29] Strategies, such as short breaks, consuming food, glucose and water can help safeguard against decision fatige,[24,30-35] something that in other industries, such as aviation, has been recognisied.[34-35]

This is not the case for healthcare, however. On the one hand, the World Health Organisation[36] recognises general fatigue as a leading contributor to medical error, and European Working Time Directive[37] restricts excessive night work and working hours. On the other hand, the type of fatigue that arises because of intensity and complexity of workload during working hours has not received the same level of recognition; despite healthcare being fraught with examples of intense cognitive work.[38-40] To-date, the impact of DM fatigue has not been explored in healthcare settings; our objective was to examine this concept for the first time within the current study design.

One way of testing and evaluating the concept of DM fatigue with an MDT is to apply the principles of 'team audit and feedback' - a process of providing non-punitive and actionable feedback to professionals to allow them to self-assess and adjust their performance, thus stimulating desired behaviour change.[41-43] Such approach was found effective in improving practice and supporting quality improvements, and can be used to aid implementation of evidence-based interventions.[41] Within our study, this approach allowed us to elicit inputs from all team members, which we then used to co-design interventions to best meet the needs of the team in addressing DM fatigue. As a team-centred approach to intervention development, implementation and evaluation, this is, to the best of our knowledge, yet to be applied to cancer MDTs.

Aim and objectives

The overarching aim of our study was to identify and co-design quality-improving team interventions (in feedback sessions) and test their effectiveness (in team audits) with an MDT with high workload and prolonged meetings.

Within this overarching aim, we had two specific objectives based on the challenging circumstances the team was in with long meetings and high workload, and the scientific knowledge-base on fatigue that can arise in such challenging circumstances.[23-35] It was therefore reasonable to explore in such concrete setting (1) the presence and impact of DM fatigue on team performance in MDT meetings, and (2) the impact of a short break in MDT meetings as a countermeasure of DM fatigue.

For peer review only - http://bmjopen.bmj.com/site/about/guidelines.xhtml

METHODS

Study design

This was a longitudinal prospective observational study carried out over a 2-year period with a breast cancer MDT. Interventions were introduced within a single arm pre-post study design in order to allow us to identify and co-design interventions (in feedback session), and test whether these interventions work under difficult real-life circumstances where workload is high and meetings exceptionally long (in team audit).

Patient and public involvement

Patients and public were not involved in the development and design of this study.

Setting

A breast cancer MDT was recruited between 2013 and 2015 from a teaching hospital of the London (UK) metropolitan area.

Participants

Participants were 15 members of a breast cancer team, and a total of 1,335 breast cancer patients reviewed at 30 MDT meetings. Availability sampling was used to identify the team with a criterion for the study being a cancer MDT from the UK National Health Service (NHS) that represents one of the most common types of cancer, and experiences high workload with prolonged meeting duration (>1h). Sample size in terms of number of MDT meetings per study phase (n=10) was determined based on our feasibility study,[1] and a prior study of our group in urology with similar workload.[12] The study was granted Ethical Approval by the local ethics committee (JRCO REF. 157441).

Intervention Design: Audit and Feedback Cycles

Interventions were co-designed and evaluated based on the principles of team audit and feedback.[41-42] In what follows, we outline what this process entailed.

Audit cycles focused on collecting observational data of team DM processes across 3 phases. In **phase 1** (baseline; MDT meetings 1 to 10; July to Nov 2013), we did not introduce any interventions, but observations of care as usual. The descriptive data from this phase have been reported as a pilot study to establish feasibility of the measurement.[1] In **phase 2** (MDT meetings 11 to 20; Feb to April 2014), we introduced two interventions including (1) *change of the room*

For peer review only

layout from lecture theatre style to a U-shape where team-members were able to face each other, and (2) formal appointment of an *MDT meeting chair-person*. The rationale for these interventions was that the change of the room layout will be more conducive to team interactions, while appointment of the formal chair will help facilitate the overall flow of the meeting and individual patient discussions. In the final **phase 3** (MDT meetings 21 to 30; Sept 2014 to March 2015), we introduced a 10-minute long break for tea, coffee and snacks halfway through the MDT meetings, i.e., typically at the 90-minute mark, which was hypothesised to help counteract negative effects of DM fatigue.

Feedback sessions focused on identifying and co-designing interventions. The interventions were identified and chosen based on the observational data from each phase, MDT recommendations, guidelines and evidence-base, as well as on team discussion and consensus within each feedback session. I.e. in each feedback session, the data from previous phase was presented to the team. The data was then benchmarked against previous observational phase, guidelines, recommendations and evidence base for cancer MDTs. In the light of this information, we discussed potential evidence-based interventions that were most appropriate and acceptable to the entire team by reaching a consensus.

More specifically, the feedback sessions occurred at 3 time points at the end of each audit phase – in June 2014, May 2014 and June 2015. Each session was allocated a 1h slot as part of the MDT meeting where we **(a)** fed back the summary of the analysis (20-minutes), **(b)** facilitated team-based review of the findings and what they meant for the team (20-minutes); and **(c)** shortlisted evidence-based interventions the team were willing to introduce into their work in the coming study period (20-minutes).

The process of implementing interventions was agreed upon in the feedback sessions, and it was facilitated/enabled in a collaborative manner. Specifically, following each feedback sessions, the research team produced minutes and actions that were approved and emailed to the MDT by their lead, a Consultant Breast Surgeon (TG). The MDT was invited to comment and identify date for intervention implementation. The task of leading the introduction/implementation of the interventions was assigned to the MDT lead. Interventions were introduced and allowed a 'bed-in' period of approximately 3 months, during which no assessments were carried out to allow the team to familiarise themselves with the novel way of working. This approach was designed at the

For peer review only - http://bmjopen.bmj.com/site/about/guidelines.xhtml

request of the MDT who needed the 'bed-in' time to ensure they did not feel they were being 'examined' by the research team at a time when they were in a state of change. The implementation process was led by the MDT, therefore.

Materials

We used a validated quantitative observational assessment tool, namely the Metric for Observation of Decision-making, (MDT-MODe; Figure 1),[10] which was tested for feasibility in our pilot study.[1] The tool has been used previously to assess various cancer MDT meetings and has shown good validity and reliability (on individual variables and composite scores).[1-4,10-14]

The MDT-MODe captures the following aspects in a meeting:

1) *Quality of presented patient information*, which includes 6 individual variables scored on a behaviourally anchored 5-point scale, namely, patients' case history, radiological images, histopathology, psychosocial issues, co-morbidities and their views on treatment options. The sum of the scores for all 6 variables represents overall quality of presented information for a patient with the higher scores indicating better quality.

2) *Quality of disciplinary contribution to patient-reviews*, which includes 6 individual variables scored on a behaviourally anchored 5-point scale, representing the surgeons, oncologists, radiologists, histopathologists, BCNs and the chair-person. The sum of the scores for all 6 variables represents overall quality of disciplinary contribution for a patient with the higher scores indicating better quality.

Figure 1

Assessor training

Prior to the formal scoring during the study, the evaluator (Cancer Nurse Specialist, SM) was trained in the use of the MDT-MODe,[10] which is a general principle for instruments assessing human factors in clinical environments.[44] Training was delivered by our team and it involved: (1) explanation of the domains, scales and their anchors, (2) background reading of peer-reviewed literature on the tool, and (3) calibration of scoring against an expert evaluator (TS) via scoring a set of pre-recorded MDT videos.

For peer review only

To ensure reliability in the use of the tool, a cross-section of the data was double-rated blindly by trained clinical (SM) and psychologist (TS) observers. To minimise H*awthorne effect*, i.e., teams changing their usual behavior due to being observed, the main study evaluator was the Cancer Nurse Specialist, the presence of whom within an MDT meeting is natural. During data collection, each evaluator was blind to the other evaluators' observations, and the observer (SM) did not participate in the MDT meetings clinically. Proficiency in scoring was set as an achievement of inter-assessor reliability of 0.70 or higher between the trainee and expert assessor; [44] this was met.

Statistical methods and variables

There were two independent variables (IVs) in the study:

- ❖ IV1 was defined as the '*study phase'* with 3 levels (phases 1, 2 and 3) in the one-way multivariate analysis, and 2 levels (phases 2 and 3) in the two-way multivariate analysis;
- ❖ IV2 was defined as the '*time lapse'* with 2 levels, namely, 1st and 2nd half of the meeting.

There were two dependent variables (DVs):

- ❖ DV1 is *quality of presented patient information* to the team as measured by MDT-MODe,[10,1]
- ❖ DV2 is *quality of disciplinary contributions to patient-review* as measured by MDT-MODe[10,1]

Three sets of analyses were conducted:

1. *Intra-class correlation coefficient (ICC)* analysis was used to assess reliability of evaluations in each phase. ICCs can range between 0 and 1, with higher values indicating better agreement.
2. *Multivariate analysis of variance (MANOVA*) was used to assess:
    a. *between-intervention differences* in DM where the effect of co-designed interventions across all 3 phases is explored using a *one-way MANOVA* with *post-hoc tests*;
    b. *within-meeting differences* in DM where presence of DM fatigue and effect of a 10-min break in phases 2 and 3 is explored using *two-way MANOVA* with *simple main effects*.
3. *Correlation analysis* was used to ascertain presence of DM fatigue across all 3 phases.

All analyses were carried out using SPSS® version 20.0. All pairwise comparisons are reported with Bonferroni-adjusted *p*-values.

For peer review only

RESULTS

Meeting characteristics

The sample consisted of overall 1,335 patients managed across the 3 study phases (see Table 1). All case-reviews for the duration of the study were conducted in the context of the set interventions. It is evident that the total number of patients discussed per phase steadily increased as the study progressed, which suggests increasing workload for the team over time.

Table 1

Reliability of evaluations

Agreement between evaluators was assessed on a subset of patient-reviews within each phase. The selection was driven predominantly by the pragmatic considerations and the availability of the second assessor who was not a member of the participating MDT and was blinded to the patient list for the meetings and the first assessor's scores.

We used single measures interclass correlation with the two-way mixed effects model and an absolute agreement definition. High reliability was obtained within each of the phases:

- *Baseline/Phase 1*: information r = 0.89, contribution r = 0.82, n = 116, 34% of the cohort;
- *Phase 2*: information r = 0.92, contribution r = 0.95, n = 116, 25% of the cohort;
- *Phase 3*: information r = 0.88, contribution r = 0.79, n = 131, 25% of the cohort.

Between-intervention differences in decision-making across all 3 phases

A one-way MANOVA was run on the dataset[45] to address the overarching aim of the study i.e. to examine effectiveness of co-designed interventions across all 3 study phases.

Specifically, a one-way MANOVA was run to determine effect of co-designed interventions (IV1 with 3 levels: phases 1, 2 and 3) on the information (DV1) and contribution (DV2) scores of the MDT-MODe.[10] Data are expressed as mean ± standard deviation. To preserve statistical power, Bonferroni adjusted p-level of 0.025 was used.

Information scores were similar between phase 1, 2 and 3 (16.31±3.71; 15.76±2.98 and 15.97±3.77, respectively), while the *contribution scores* were lower in phase 1 than 2 and 3 (17.16±3.23; 22.13±3.40; 18.81±5.50, respectively). There was statistically significant difference between the intervention phases on the *combined DVs*, p<0.001.

For peer review only

For peer review only - http://bmjopen.bmj.com/site/about/guidelines.xhtml

Follow-up univariate ANOVAs showed that the *information scores* (**Figure 2a**) alone **did not** reveal significant differences between phases (p=0.09), while the *contribution scores* **did** (p<0.025). Bonferroni post-hoc tests revealed that for *contribution scores* (**Figure 2b**), phase 2 had significantly higher mean score than phases 1 (p<0.02) and 3 (p<0.02); and that phase 3 had significantly higher mean score than phase 1 (p<0.02). **See figure 2a and b** for a graphical representation of the results.

In sum, the findings show that the *quality of information* remained largely similar across phases, while the *quality of contribution* improved in phases 2 and 3 relative to phase 1 but with no linear improvement across phases.

Figure 2

Within-meeting differences in decision-making in phases 2 and 3

A two-way MANOVA was run on the dataset[45] to address the two objectives in our study i.e. (1) the presence and impact of DM fatigue on team performance in MDT meetings, and (2) the impact of a short break in MDT meetings as a countermeasure of DM fatigue.

Specifically, a two-way MANOVA was conducted to examine interaction effects between *IV1 or a 10-minute break* (two levels: phase 2 meetings with no break, and phase 3 meetings with a break), and IV2 or '*time lapse'* (two levels: 1st and 2nd half of meetings) on the information (DV1) and contribution (DV2) scores of the MDT-MODe.[10] Data are expressed as mean ± standard deviation.

There was a significant interaction effect between *10-minute break* and *time lapse* on the *information* (p<0.01) and *contribution scores* (p<0.001). An *analysis of simple main effects* for a *10-minute break* and *time lapse* was performed with significance Bonferroni-adjusted for p<0.0125. **See figure 2c and d** for a graphical representation of the results reported below.

Mean information scores for 1st and 2nd half of meetings were 16.36±2.49 and 15.10±3.34 in phase 2, and in phase 3 they were 16.00±3.96 and 15.94±3.61, respectively. There was a significant difference in mean *information scores* for 1st v. 2nd half of the meeting in phase 2 (p<0.001) and a non-significant difference in phase 3 when the meeting break was introduced (p=0.845). Mean *information score* (**Figure 2c**) in phases 2 and 3 did not significantly differ in the 1st half of the meeting, 0.36 (95% CI, -0.24 to 0.96) p=0.238; however, in the 2nd half of the meeting, mean

For peer review only - http://bmjopen.bmj.com/site/about/guidelines.xhtml

information score was significantly higher in phase 3 than phase 2, -0.84 (95% CI -1.45 to -0.24), $p<0.01$.

Mean *contribution scores* for 1st and 2nd half of the meeting were 22.67±2.83 and 21.52±3.87 in phase 2, and in phase 3 they were 17.66±5.35 and 19.85±5.43, respectively. There was also a significant difference in mean *contribution scores* for 1st v. 2nd half of the meeting in phase 2 ($p<0.01$), and also in phase 3 ($p<0.001$). In phase 2, mean *contribution score* (**Figure 2d**) was significantly higher in the 1st as opposed to the 2nd half of the meeting, 1.15 (95% CI, 0.32 to 1.98), $p<0.01$, and in phase 3, the mean was significantly lower in the 1st as opposed to the 2nd half of the meeting, -2.19 (95% CI, -2.97 to -1.41), $p<0.001$.

In sum, *quality of information and contribution* was reduced in the 2nd half of the meeting when the MDT did not have a 10min break (phase 2). In contrast, when the MDT had a break (phase 3), the quality of information remained unchanged, while the quality of contribution improved.

Correlation analysis: ordinal position of cases and quality of dm across study phases

A follow-up analysis was conducted on the ordinal position of cases within meetings, and information and contribution scores *to ascertain* performance decrements across all 3 phases, and improvements obtained in phase 3 because of a 10-minute break. Ordinal position of a case within an MDT meeting is taken as an indicator of potential effects of DM fatigue: the later a case is reviewed during the MDT meeting, the more cases the team would have reviewed in a sequential manner prior to it.

Table 2 shows significant negative correlations between ordinal position of cases, and contribution and information scores in phases 1 and 2 - i.e., as the ordinal position of cases increases (i.e., the patient is reviewed later in the meeting), the information and contribution scores decrease (i.e., team interaction and clinical input measures worsen). In phase 3, however, when the short break was introduced, both coefficients are non-significant, indicating overall improvement – i.e., a lack of impact of the repetitive DM process on the team interaction and clinical input indicators.

Table 2

Table 2 also shows that the intervention package introduced in phase 2 (change of room layout and appointing a meeting-chair) did not influence the quality of DM when assessed within

For peer review only - http://bmjopen.bmj.com/site/about/guidelines.xhtml

meetings; these effects are only detectable in the between-intervention analysis (see Figure 2a and 2b for a graphical representation of these effects).

Team's feedback on the conduct of the meetings

In the final feedback session (June 2015), the team recognised that the meeting break and seating rearrangement were useful and had positive impact on their working, while appointing a rotating chairperson presented with challenges and is something that would need more focus in order to ensure consistency across weekly meetings. The team reported two reasons for this, one, team friction and lack of clarity around who is chairing, and second, fatigue that the chairperson experiences by having to chair the meeting and contribute clinically to discussion ('chairing fatigue'). The team proposed that, going forward, this could be addressed by assigning the chairing role to another member of the team in the 2nd half of the meeting.

Hence while the fidelity of intervention delivery was good throughout – in particular for the meeting break and change of room layout which were implemented as agreed/planned in the feedback sessions, appointing a meeting chair was more challenging as it appears that although a rotating chair was appointed throughout, due to team friction, not all appointed chairs were accepted by other members of the team in the same manner.

DISCUSSION

The overall aim of this study was to examine effectiveness of co-designed interventions with a breast cancer team with high workload and prolonged meeting duration, and within this, explore presence and impact of DM fatigue, and a short break as a countermeasure. Our findings were threefold. Firstly, our study lends support for the concept of DM fatigue in MDT meetings.[23-24] In phase 2, the information and contribution quality were significantly lower in the 2nd v. 1st half of the meeting. The serial position of cases in the meetings in phases 1 and 2 were also negatively correlated with information and contribution quality, indicating performance decrements as meetings progressed. Secondly, our study lends support to a premise that short break in the middle of a meeting can counterbalance the effect of DM fatigue.[24,30-35] For instance, after the break was introduced in phase 3, serial position of cases no longer showed significantly negative correlation with information and contribution quality, and the scores in the 2nd half of the meeting no longer showed significant decrease.

For peer review only - http://bmjopen.bmj.com/site/about/guidelines.xhtml

Thirdly, we found a significant increase in information and contribution quality after the introduction of co-designed interventions in phases 2 and 3 in comparison to baseline (or, phase 1). This somewhat lends support to co-designed interventions via audit and feedback.[41-43] However, a significant decrease was evident in phase 3 in comparison to phase 2, pointing to challenges at sustaining initially implemented interventions over time. In line with the final team's feedback, one explanation may be chairing fatigue and team friction, which highlights the need for continuous quality improvements and implementation science approaches to help improve our understanding of barriers and facilitators to the uptake of evidence-based interventions for cancer MDTs. It is possible that the feedback should be provided to the team at shorter intervals (after every 5th as opposed to every 10th meeting) to help reinforce the agreed change and goals. Another element that could have (also) indirectly contributed to these findings is the steady increase in workload across phases (Table 2), which is known to negatively impact MDT-working.[16-17]

Nonetheless, despite the nonlinear trajectory between phases 2 and 3, the improvements were made in the within-meeting performance i.e. between 1st and 2nd half of the meeting in phase 3 after the 10-minute break was introduced. This lends support to the concept of DM fatigue - i.e., fatigue that arises because of consecutive cognitive efforts in formulating treatment recommendations, previously explored in other fields (e.g. judicial DM).[24-25] Improved quality of discussion between different disciplines is observed when break is introduced with the quality of presented patient information becoming more stable throughout the meeting. What is more, the 10-minute break did not add additional time to the meeting duration (Table 1), indicating that taking a break made the team more time efficient. The concept of DM fatigue has not yet been explored within cancer MDT meetings, and to our knowledge this is the first study of its kind, with implications for the way meetings are currently structured.

Implications

The implications for meeting structure are far-reaching. It is not only the number of hours worked in a 24-hour period, but also the number of consecutive hours, including the type, intensity and complexity of a task, a clinician engages in without adequate break that requires more focus and recognition. Healthcare is a highly demanding work setting, and apart from MDT meetings, there are many examples of cognitively intense settings, including for e.g., ward rounds and intensive care units.[38-39] While the general health worker fatigue is addressed by the European Working Time Directive[37] which restricts excessive night work and working hours, the type of fatigue that

For peer review only - http://bmjopen.bmj.com/site/about/guidelines.xhtml

arises as a result of intensity and complexity of the workload during the working hours is not adequately acknowledged or safeguarded with recommendations, such as a short break, for instance. It is understood however that the fatigue is a leading contributor to medical error and injury,[36] and that intense episodes of workload in healthcare are on the increase,[19-21,5] as clinical teams are trying to maximise productivity in the face of severe staff shortages[22] and financial pressures.[20-21]

Limitations

Our findings need to be interpreted within certain limitations (some of which have been previously reported).[1]

First, participants in our study were aware that they were being observed. This was necessary due to (a) the methodological approach undertaken in our study, i.e. team audit and feedback that requires the results to be fed back to the team and interventions co-designed thus making the research useful to the team, as well as (b) the ethical and regulatory constraints which meant that we had to provide full description of the study to the participants – this is due to the importance of informed consent (in line with the Good Clinical Practice), and the absence of such consent i.e. deception (e.g. where MDT members are not aware that they are being observed) being regarded as high-risk to participants, requiring checks and considerations by the research ethics committee that reviewed current study (where MDT members knew that they were being observed; under JRCO REF. 157441). Hence, we cannot rule out *Hawthorne effect* and the observer bias. While the former is a natural limitation to observational studies, we ensured that the main study evaluator was a clinician, in our case, Cancer Nurse Specialist, the presence of whom within an MDT meeting is natural. In terms of the latter, we used a validated tool with a subset of cases scored by *trained* evaluators in pairs who were blind to one another's observations within each phase of the study.

Second, while this is a large-scale study for its nature (observations in real-time), we acknowledge that there are cancer MDT meetings that are not as long as the ones reported here, hence the *generalizability of our findings may be limited* to MDTs with high workloads and prolonged meeting duration within the NHS setting. However, the global economic and healthcare landscape is rapidly changing – i.e. cancer incidence[19-20] is on the increase, as well as MDT workload,[5,19] financial pressures,[20-21] and staff shortages.[22] The findings that we report may therefore become

For peer review only

increasingly relevant to MDTs across different tumor types (and other healthcare settings) globally and could be profitably explored to determine the extent to which they apply to them.

Third, our study is of *pre-post design*, which can limit generalizability of our findings. This is because there is no control over other (extraneous) elements that are also changing at the same time as the intervention is implemented. While we understand that randomized controlled trials provide increased control of such extraneous factors allowing better precision in testing the efficacy of interventions, the aim of our study was to examine the effectiveness of interventions that were identified and co-designed with the participating team under the challenging real-world circumstances where workload and meeting duration are exceptionally high. Nonetheless, future research could adopt an RCT approach to testing the co-designed interventions identified as part of our study with multiple different MDTs to ascertain the impact of each on team functioning under ideal controlled circumstances, which in combination with our effectiveness findings with a single team under real-life circumstances would greatly enhance generalisability. However, MDTs tend to have rather different problems and priorities,[46] and so if they opt for a co-designed approach, they may end up with different interventions. Hence one would need to start off with a few smaller scale studies, such as the current one, followed by a wider consensus exercise across MDTs where a selection of team and functional improvement interventions could be identified and prioritised – these could then be designed into a randomised controlled trial.

The strength of our methodological approach resides in a large sample size (*N*=1335), a robust methodology with validated tools and training, and an approach to improvement that is highly team-centred/driven, engaging, inclusive, non-intrusive and feasible for the team (i.e. does not add to their workload). Such approach has allowed us to capture complex organisational behaviour of the MDT in real time, providing good external validity, evidence of effectiveness, while identifying a set of acceptable co-designed interventions for MDTs with high workload and increased meeting duration.

Fourth, the validated tool used in the current study (MDT-MODe) *does not allow for individual person-level assessment*; only disciplinary group-level with the unit of analysis being a case-discussion (and not an individual team member; Figure 1). Such approach has advantages when evaluating a relatively small (single) team because it ensures team safety by minimizing the risk of defensive routine and blaming a particular team member for performance difficulties which could in turn distract the team from addressing their performance problems constructively.[47] We

For peer review only - http://bmjopen.bmj.com/site/about/guidelines.xhtml

acknowledge however that such approach also has limitations because it does not capture (the effect of) team interaction, as well as (the effect of) individual team member's level of seniority, experience, and personality, and so the effect of the physician versus the team, or style of presentation of different radiologists/histopathologists cannot be accounted for. To address these questions, a different methodological approach may be better suited, such as Conversation Analysis for instance, which allows for an in-depth analyses of team interaction on an individual person-level. Also, development of tools for MDTs should take this limitation into account.

Lastly, while the current study is focused on DM process at the point of the MDT meeting, we have not linked these processes to clinical, patient-related outcomes. As a result, the safety implications of this analysis remain exploratory and are not yet equated to clinical outcomes.

Further research

The objective of our study was to investigates presence and impact of fatigue on *DM processes* in a team with high workload; as such, we did not address how it impacts the *quality of decisions reached* (e.g., their clinical suitability for the patient) or *patient outcomes*. This is however an important next step that should be further explored in the light of our findings and previous research showing that DM fatigue leads to impulsive decisions, status quo, and reduced ability to effectively evaluate information – these could potentially have a knock-on effect on patient outcomes.[17-22] Further research is also needed to assess presence of DM fatigue across different cancer MDTs, particularly those with high workloads, and explore effectiveness of various evidence-based cognitive strategies.[24,30-33] Efforts should be channelled toward safeguarding optimal DM in MDT meetings, taking into account the intensity and complexity of the workload, with strategies in place as standard practice - such as, for instance, a maximum limit of cases allowed for a single meeting, mandatory short break (as practiced in the aviation industry), and trained team lead/chairperson to help the team effectively navigate through workload.[6] Team-centred, co-designed approaches may prove useful in helping identify appropriate (tailored) strategies for a team, however, challenges exist at sustaining change over time; hence, a need for continuous quality improvement and implementation science approaches in the field of cancer MDTs.

CONCLUSIONS

Previous research has shown variability in the quality of DM across cancer MDT meetings, with internal factors, such as group composition and leadership, and external circumstances, such as

For peer review only

increased workload, time pressures and changing economic landscape held accountable. Our study demonstrates for the first time that quality of DM in cancer MDT meetings grows worse during consecutive cognitive efforts and is positively influenced with a break. Using principles of team audit and feedback to co-design team-centred interventions is a useful approach in helping initiate improvements, however, challenges exist at sustaining interventions over time. Building on our findings, further research in MDTs is needed to investigate effects of DM fatigue on the quality of decisions reached and patient outcomes, ascertain its presence across different cancer teams, and encourage implementation of quality-improving strategies to protect optimal DM. The work could be extrapolated to other areas of clinical (and non-clinical) practice and may have implications for other areas that have equally intense periods of cognitively demanding work.

ACKNOWLEDGEMENTS

The authors would like to thank the breast cancer MDT and their members for their time and commitment to this project.

For peer review only

DISCLOSURE

Nick Sevdalis is the Director of London Safety & Training Solutions Ltd, which provides team working, patient safety and improvement skills training and advice on a consultancy basis to hospitals and training programs in the UK and internationally. James Green is a Director of Green Cross Medical Ltd that developed MDT FIT for use by National Health Service Cancer Teams in the UK. The other authors have no conflicts of interest to report.

FUNDING

Financial support for this study was provided entirely by the UK's National Institute for Health Research (NIHR) via the Imperial Patient Safety Translational Research Centre. Sevdalis and Soukup's research is funded by the NIHR via the 'Collaboration for Leadership in Applied Health Research and Care South London' at King's College Hospital NHS Foundation Trust, London, UK. Sevdalis is also a member of King's Improvement Science, which is part of the NIHR CLAHRC South London and comprises a specialist team of improvement scientists and senior researchers based at King's College London. Its work is funded by King's Health Partners (Guy's and St Thomas' NHS Foundation Trust, King's College Hospital NHS Foundation Trust, King's College London and South London and Maudsley NHS Foundation Trust), Guy's and St Thomas' Charity, the Maudsley Charity and the Health Foundation. The funding agreement ensured the authors' independence in designing the study, interpreting the data, writing, and publishing the report. The views expressed are those of the authors and not necessarily those of the NHS, the NIHR or the Department of Health and Social Care.

AUTHORS' CONTRIBUTIONS

In line with the guidelines by the International Committee of Medical Journal Editors, all authors for this study (i.e., TS, TG, SM, JG, and NS) have made substantial contributions to conception and design, or acquisition of data, or analysis and interpretation of data; have been involved in drafting the manuscript or revising it critically for important intellectual content; have given final approval of the version to be published; and have agreed to be accountable for all aspects of the work in ensuring that questions related to the accuracy or integrity of any part of the work are appropriately investigated and resolved.

For peer review only

DATA SHARING STATEMENT

The anonymised dataset supporting this study is available on Zenodo, a research data repository, under the Creative Commons Attribution Non-Commercial Non-Derivative 4.0 license. The researchers are free to reuse and redistribute the data set on the condition that they *attribute it*, that they *do not use it for commercial purposes*, and that they *do not alter it*. For any reuse or redistribution, researchers must make clear to others *the license terms* of this work and cite the dataset accordingly.

For peer review only

REFERENCES

1 Gandamihardja TAK, Soukup T, McInerney S, Green JSA, Sevdalis N. (in press) Analyzing breast cancer multidisciplinary patient management: a prospective observational evaluation of team clinical decision making. *World J Surg* 2018;in press.

2 Sarkar S, Arora S, Soukup T, Lamb BW, Shah S, Green JSA, Sevdalis N, Darzi, A. A multi-centre study evaluating performance of multidisciplinary teams: Urology vs the top cancer killers. *Eur Urol Suppl* 2014;13: e878-e878a.

3 Lamb BW, Sevdalis N, Benn J, Vincent C, Green JS. Multidisciplinary cancer team meeting structure and treatment decisions: A prospective correlational study. *Ann Surg Onc* 2013;20: 715–722.

4 Jalil R, Akhter W, Lamb BW, Taylor C, Harris J, Green JSA. Validation of team performance assessment of multidisciplinary tumor boards. *J Urol* 2014;192(3): 891-898.

5 Cancer Research UK. Improving the effectiveness of multidisciplinary team meetings in cancer services. London, Cancer Research UK; 2017.

6 National Cancer Action Team. The characteristics of an effective multidisciplinary team (MDT). London, UK: National Cancer Action Team; 2010.

7 Department of Health. Manual for Cancer Services. London, UK: The Department of Health; 2004.

8 Raine R, Xanthopoulou P, Wallace I. Determinants of treatment plan implementation in multidisciplinary team meetings for patients with chronic diseases: a mixed-methods study. *BMJ Qual Saf* 2014;23: 867–876.

9 Hong NJ, Wright FC, Gagliardi AR, Paszat LF. Examining the potential relationship between multidisciplinary cancer care and patient survival: An international literature review. *J Surg Oncol* 2010;102: 125-134.

10 Lamb BW, Wong HWL, Vincent C, Green JSA, Sevdalis N. Teamwork and team performance in multidisciplinary cancer teams: Development of an observational assessment tool. *BMJ Qual Saf* 2011;20: 849-856.

11 Lamb BW, Brown K, Nagpal K, Vincent C, Green JS, Sevdalis N. Quality of care management decisions by multidisciplinary cancer teams: A systematic review. *Ann Surg Oncol* 2011;18: 2116-2125.

For peer review only - http://bmjopen.bmj.com/site/about/guidelines.xhtml

12 Lamb BW, Green JS, Benn J, Brown KF, Vincent C, Sevdalis N. Improving decision making in multidisciplinary tumor boards: Prospective longitudinal evaluation of a multicomponent intervention for 1,421 patients. *J Am Coll Surg* 2013;217(3): 412-420.

13 Soukup T, Lamb BW, Sarkar S, Arora S, Shah S, Darzi A, Green JSA, Sevdalis N. Predictors of treatment decision in multidisciplinary oncology meetings: A quantitative observational study. *Ann Surg Oncol* 2016;23(13): 4410-4417.

14 Soukup T, Petrides KV, Lamb BW, Sarkar S, Arora S, Shah S, Darzi A, Green JSA, Sevdalis N. The anatomy of clinical decision-making in multidisciplinary cancer meetings: A cross-sectional observational study of teams in a natural context. *Medicine* 2016;95;e3885.

15 Stairmands J., Signal L, Sarfati D, Jackson C, Batten L, Holdaway M, Cunningham C. Consideration of comorbidity in treatment decision-making in multidisciplinary team meetings: A systematic review. *Ann Oncol* 2015;26(7): 1325-1332.

16 Kidger J, Murdoch J, Donovan JL, Blazeby JM. Clinical decision-making in a multidisciplinary gynaecological cancer team: a qualitative study. *BJOG* 2009;116(4): 511-517.

17 Haward R, Amir Z, Borrill C, Dawson J, Scully J, West M, et al. Breast cancer teams: The impact of constitution, new cancer workload, and methods of operation on their effectiveness. *Br J Cancer* 2003;89(1): 15-22.

18 Poole MS, Hollingshead AB. Theories of small groups: Interdisciplinary perspectives. Thousand Oaks, CA: Sage; 2005.

19 Mistry M, Parkin DM, Ahmad AS, Sasieni P. Cancer Incidence in the UK: Projections to the Year 2030. *Br J Cancer* 2011;105: 1795-1803.

20 World Health Organization. World Cancer Report 2014. France: International Agency for Research on Cancer, World Health Organization; 2014.

21 NHS England. Everyone Counts: Planning for Patients 2014/2015 to 2018/2019. England, UK: NHS England; 2014.

22 NHS Improvement. Evidence from NHS Improvement on clinical staff shortages: A workforce analysis. London, NHS Improvement; 2016.

23 Harewood GC, Chrysostomou K, Himy N, Leong WL. Impact of operator fatigue on endoscopy performance: Implications for procedure scheduling. *Dig Dis Sci* 2009;54(8): 1656–1661.

For peer review only

24 Danziger S, Levav J, Avnaim-Pesso L. (2012). Extraneous factors in judicial decisions. *Proc Natl Acad Sci* 2012;108(17): 6889-6892.

25 Kool W, McGuire JT, Rosen ZB, Botvinick MM. Decision making and the avoidance of cognitive demand. *J Exp Psychol* 2010;139(4): 665-682.

26 Eidelma S, Crandall CS. Bias in favour of the Status Quo. *Soc Personal Psychol Compass* 2012;6(3): 270-281.

27 Weissman DH, Roberts KC, Visscher KM, Woldorff MG. The neural bases of momentary lapses in attention. *Nat Rev Neurosci* 2006;9: 971-978.

28 Kaplan S, Berman MG. Directed attention as a common resource for executive functioning and self-regulation. *Perspect Psychol Sci* 2010;5: 43-57.

29 Sawin DA, Scerbo MW. Effects of instruction type and boredom proneness in vigilance: Implications for boredom and workload. *Hum Factors* 1995;37(4): 752-765.

30 Tyler JM, Burns KC. After depletion: The replenishment of the self's regulatory resources. *Self Identity* 2008;7: 305–321.

31 McMahon AJ, Scheel MH. Glucose promotes controlled processing: Matching, maximising and root beer. *Judgm Decis Mak* 2010;5(6): 450-457.

32 Kempton MJ, Ettinger U, Foster R, Williams SC, Calvert GA, Hampshire A, et al. Dehydration affects brain structure and function in healthy adolescents. *Hum Brain Mapp* 2011;32(1): 71-9.

33 Hagger MS, Chatzisarantis NL. The sweet taste of success: the presence of glucose in the oral cavity moderates the depletion of self-control resources. *Pers Soc Psych Bull* 2013;39: 28–42.

34 Neri DF, Shappell SA, DeJohn CA. Simulated sustained flight operations and performance, Part 1: Effects of fatigue. *Mil Psychol* 1992;4(3): 137-155.

35 Neri DF, Oyung RL, Colletti LM, Mallis MM, Tam PY, Dinges DF. Controlled breaks as a fatigue countermeasure on the flight deck. *Aviat Space Environ Med* 2002;73(7): 654-664.

36 World Health Organisation. WHO Patient Safety Curriculum Guide for Medical Schools. France, A World Alliance for Safer Health Care; 2009.

For peer review only

For peer review only - http://bmjopen.bmj.com/site/about/guidelines.xhtml

37 The European Parliament and the Council of the European Union. Directive 2003/88/EC of the European Parliament and of the Council of 4 November 2003 concerning certain aspects of the organisation of working time. *Official Journal of the European Union*; 2013.

38 Reader TW, Cuthberson BH, Decruyenaere J. Burnout in the ICU: Potential consequences for staff and patient well-being. *Intensive Care Med* 2008;34(1): 4-6.

39 Reader TW, Reddy G, Brett SJ. Impossible decision? An investigation of risk trade-offs in the intensive care unit. *Ergonomics* 2017;61: 122-133.

40 Reid PP, Compton D, Grossman JH, Fanjiang G. Building a better delivery system: A new engineering/health care partnership. Washington, D.C.: The National Academies Press; 2005.

41 Ivers N, Jamtvedt G, Flottorp S, Young JM, Odgaard-Jensen J, French SD. Audit and feedback: effects on professional practice and health care outcomes, Cochrane Database Sys Rev, no. 2, CD000259; 2006.

42 Flottorp SA, Jamtvedt G, Gibis B, McKee M. Using audit and feedback to health professionals to improve the quality and safety of health care. Denmark: World Health Organization on the behalf of the European Observatory on Health Systems and Policies; 2010.

43 Michie S, Johnston M, Abraham C, Lawton R, Parker D, Walker A. Making psychological theory useful for implementing evidence-based practice: a consensus approach. *Qual Saf Health Care* 2005;14: 26-33.

44 Hull L, Arora S, Symons NR, Jalil R, Darzi A, Vincent C. Delphi expert consensus panel. Training faculty in nontechnical skill assessment: national guidelines on program requirements. *Ann Surg* 2013;258(2): 370-375.

45 Soukup, T. Longitudinal intervention in a breast cancer team [Data set]. Zenodo 2018; http://doi.org/10.5281/zenodo.582198

46 Soukup T, Lamb BW, Arora S, Darzi A, Sevdalis N, Green JSA. Successful strategies in implementing a multidisciplinary team working in the care of patients with cancer: An overview and synthesis of the available literature. *J Multidisc Healthc 2018;*11:49-61.

47 West MA. Effective teamwork: practical lessons from organisational research. John Wiley & Sons; 2012.

For peer review only - http://bmjopen.bmj.com/site/about/guidelines.xhtml

FIGURE CAPTIONS

Figure 1. Metric for the observation of decision-making in cancer multidisciplinary team meetings (MDT-MODe)

Figure 2. Mean scores for information and contribution quality across the observational phases 1, 2 and 3 (a and b), as well as across the 1st and 2nd half of the meetings in phases 2 and 3 (c and d)

For peer review only

For peer review only - http://bmjopen.bmj.com/site/about/guidelines.xhtml

TABLES

For peer review only

Table 1. Meeting characteristics of the breast cancer team across the intervention phases

**Meeting characteristics**	**Phase 1**				**Phase 2**				**Phase 3**			
	Total	Mean	Min	Max	Total	Mean	Min	Max	Total	Mean	Min	Max
Number of meetings observed*	10	-	-	-	10	-	-	-	10	-	-	-
Number of patients per meeting**	346	42	29	51	467	55	44	73	522	62	52	70
Time per patient-review (MM:SS)	-	03:20	00:31	09:00	-	03:00	00:47	09:06	-	02:06	00:10	12:49
Meeting duration (HH:MM)	-	03:05	02:45	03:30	-	03:00	02:00	03:30	-	02:53	01:30	03:25

Note. *Total *N* of meetings observed across all 3 phases = 30. **Total *N* of patients discusses across all 3 phases = 1,335.

For peer review only

Table 2. Pearson correlation between ordinal position of cases and the information and contribution scores

	**Information score**	**Contribution score**	***n***
Ordinal position of patients in **phase 1**	-0.254*	-0.160*	346
Ordinal position of patients in **phase 2**	-0.206*	-0.128*	467
Ordinal position of patients in **phase 3**	-0.078	0.072	522

Note. *$p < 0.01$. $N = 1{,}335$ patient-reviews.

For peer review only

For peer review only - http://bmjopen.bmj.com/site/about/guidelines.xhtml

MDT- MODe II

Metric for the Observation of Decision making II

Whipps Cross University Hospital NHS Trust — Building Futures Together

Imperial College London

CPSSQ

			Information							Discussion							OUTCOME	
#	Site	point	Hx	X-ray	Path	Psy/soc/	comorbid	Patient's view	Patient seen by a member	Chair	Surg	Phys*	Oncolo	Nurse	Radiolo	Histopath	Y/D/N	Observer aware of the decision
1																		
2																		
3																		
4																		
5																		

Figure 1. Metric for the observation of decision-making in cancer multidisciplinary team meetings (MDT-MODe)

218x59mm (144 x 144 DPI)

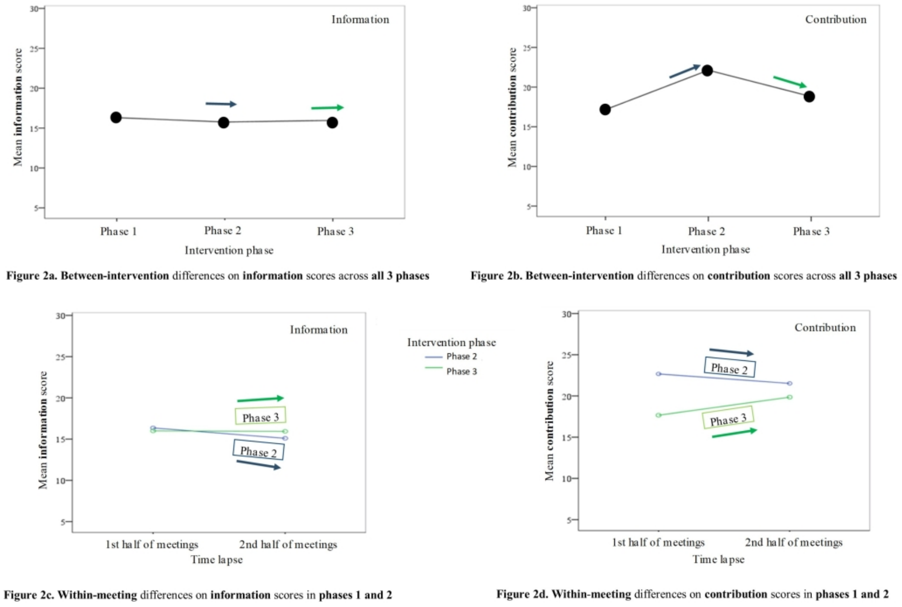

Figure 2. Mean scores for information and contribution quality across the observational phases 1, 2 and 3 (a and b), as well as across the 1st and 2nd half of the meetings in phases 2 and 3 (c and d)

297x209mm (300 x 300 DPI)

STROBE Statement

Checklist of items that should be included in reports of observational studies

Section/Topic	Item No	Recommendation	Reported on Page No
**Title and abstract**	1	(*a*) Indicate the study's design with a commonly used term in the title or the abstract	1
		(*b*) Provide in the abstract an informative and balanced summary of what was done and what was found	3
**Introduction**			
Background/rationale	2	Explain the scientific background and rationale for the investigation being reported	4-5
Objectives	3	State specific objectives, including any prespecified hypotheses	5
**Methods**			
Study design	4	Present key elements of study design early in the paper	5-6
Setting	5	Describe the setting, locations, and relevant dates, including periods of recruitment, exposure, follow-up, and data collection	6
Participants	6	(*a*) *Cohort study*—Give the eligibility criteria, and the sources and methods of selection of participants. Describe methods of follow-up<br>*Case-control study*—Give the eligibility criteria, and the sources and methods of case ascertainment and control selection. Give the rationale for the choice of cases and controls<br>*Cross-sectional study*—Give the eligibility criteria, and the sources and methods of selection of participants	6
		(*b*) *Cohort study*—For matched studies, give matching criteria and number of exposed and unexposed<br>*Case-control study*—For matched studies, give matching criteria and the number of controls per case	
Variables	7	Clearly define all outcomes, exposures, predictors, potential confounders, and effect modifiers. Give diagnostic criteria, if applicable	8
Data sources/measurement	8*	For each variable of interest, give sources of data and details of methods of assessment (measurement). Describe comparability of assessment methods if there is more than one group	7
Bias	9	Describe any efforts to address potential sources of bias	9
Study size	10	Explain how the study size was arrived at	6
Quantitative variables	11	Explain how quantitative variables were handled in the analyses. If applicable, describe which groupings were chosen and why	8
Statistical methods	12	(*a*) Describe all statistical methods, including those used to control for confounding	9
		(*b*) Describe any methods used to examine subgroups and interactions	9
		(*c*) Explain how missing data were addressed	9
		(*d*) *Cohort study*—If applicable, explain how loss to follow-up was addressed<br>*Case-control study*—If applicable, explain how matching of cases and controls was addressed<br>*Cross-sectional study*—If applicable, describe analytical methods taking account of sampling strategy	n/a
		(*e*) Describe any sensitivity analyses	n/a

For peer review only - http://bmjopen.bmj.com/site/about/guidelines.xhtml

Section/Topic	Item No	Recommendation	Reported on Page No
**Results**			
Participants	13*	(a) Report numbers of individuals at each stage of study—eg numbers potentially eligible, examined for eligibility, confirmed eligible, included in the study, completing follow-up, and analysed	9
		(b) Give reasons for non-participation at each stage	n/a
		(c) Consider use of a flow diagram	n/a
Descriptive data	14*	(a) Give characteristics of study participants (eg demographic, clinical, social) and information on exposures and potential confounders	9
		(b) Indicate number of participants with missing data for each variable of interest	n/a
		(c) *Cohort study*—Summarise follow-up time (eg, average and total amount)	n/a
Outcome data	15*	*Cohort study*—Report numbers of outcome events or summary measures over time	
		Case-control study—Report numbers in each exposure category, or summary measures of exposure	
		Cross-sectional study—Report numbers of outcome events or summary measures	10-12
Main results	16	(*a*) Give unadjusted estimates and, if applicable, confounder-adjusted estimates and their precision (eg, 95% confidence interval). Make clear which confounders were adjusted for and why they were included	n/a
		(*b*) Report category boundaries when continuous variables were categorized	n/a
		(*c*) If relevant, consider translating estimates of relative risk into absolute risk for a meaningful time period	n/a
Other analyses	17	Report other analyses done—eg analyses of subgroups and interactions, and sensitivity analyses	10-12
**Discussion**			
Key results	18	Summarise key results with reference to study objectives	12-13
Limitations	19	Discuss limitations of the study, taking into account sources of potential bias or imprecision. Discuss both direction and magnitude of any potential bias	14
Interpretation	20	Give a cautious overall interpretation of results considering objectives, limitations, multiplicity of analyses, results from similar studies, and other relevant evidence	13
Generalisability	21	Discuss the generalisability (external validity) of the study results	14
**Other Information**			
Funding	22	Give the source of funding and the role of the funders for the present study and, if applicable, for the original study on which the present article is based	1

**Give information separately for cases and controls in case-control studies and, if applicable, for exposed and unexposed groups in cohort and cross-sectional studies.*

Note: An Explanation and Elaboration article discusses each checklist item and gives methodological background and published examples of transparent reporting. The STROBE checklist is best used in conjunction with this article (freely available on the Web sites of PLoS Medicine at http://www.plosmedicine.org/, Annals of Internal Medicine at http://www.annals.org/, and Epidemiology at http://www.epidem.com/). Information on the STROBE Initiative is available at www.strobe-statement.org.

For peer review only - http://bmjopen.bmj.com/site/about/guidelines.xhtml
